# Hierarchical communities in the walnut structure of the Japanese production network

**DOI:** 10.1371/journal.pone.0202739

**Published:** 2018-08-29

**Authors:** Abhijit Chakraborty, Yuichi Kichikawa, Takashi Iino, Hiroshi Iyetomi, Hiroyasu Inoue, Yoshi Fujiwara, Hideaki Aoyama

**Affiliations:** 1 Graduate School of Simulation Studies, The University of Hyogo, Kobe, Japan; 2 Faculty of Science, Niigata University, Niigata, Japan; 3 Graduate School of Science, Kyoto University, Kyoto, Japan; Universidad Nacional de Mar del Plata, ARGENTINA

## Abstract

This paper studies the structure of the Japanese production network, which includes one million firms and five million supplier-customer links. This study finds that this network forms a tightly-knit structure with a core giant strongly connected component (GSCC) surrounded by IN and OUT components constituting two half-shells of the GSCC, which we call a*walnut* structure because of its shape. The hierarchical structure of the communities is studied by the Infomap method, and most of the irreducible communities are found to be at the second level. The composition of some of the major communities, including overexpressions regarding their industrial or regional nature, and the connections that exist between the communities are studied in detail. The findings obtained here cause us to question the validity and accuracy of using the conventional input-output analysis, which is expected to be useful when firms in the same sectors are highly connected to each other.

## Introduction

A macro economy is the aggregation of the the dynamic behaviour of agents who interact with each other under diverse external (non-economic) conditions. Economic agents are numerous and include consumers, workers, firms, financial institutions, government agencies, and countries. The interactions of these agents result in the creation of economic networks, where nodes are economic agents, and links (edges) connect agents that interact with each other. Therefore, there are various kinds of economic networks depending on the nature of the interactions, which form an overlapping multi-level network of networks. Thus, any evidence-based scientific investigation of the macro economy must be based on an understanding of the real nature of these interactions and the economic network of networks that they form. This concept also applies to the micro-level perspective of economic agents: without knowing who a firm trades with, how can anyone hope to determine the future of that firm? Therefore, it is highly important to use actual network information when studying economic dynamics with either agent-based modelling/simulations or other means of systematic studies such as determining the debt-rank of an economic agent [1–5]. Without this information, it is difficult to apply the validity of the results to the actual economy.

In this paper, we study the structure of one of the most important networks, the production network, which is formed by firms (as nodes) and trade relationships (as links) [6–9]. In the scientific study of both the macro and the micro economy, the production network of the real economic world is a topic of high importance. Before one engages in agent-model building and developing simulations, one needs to understand the structure of this network to be able to understand the dynamics of this network and eventually reach into the realm of economic fluctuations, business cycles, systemic crises, as well as firms’ growth and decline. Therefore, in the next Section, we describe the overall statistics and visualization and refer to the unique overall structure of the network as a *“walnut”* structure. This type of structure is quite different from what is expected because of the existence of the IN-giant strongly connected component (GSCC)-OUT components: In the trade network, the flow of materials and goods begins with imported/mined/harvested raw materials such as oil, iron, other metals and food. Firms who engage in this business form the IN components. These compnoents are then processed to become various products such as semiconductors or powdered food by firms, which are considered to be GSCC components, before they are made into consumer goods by firms, which are considered to be the OUT components. One might think that the existence of IN-GSCC-OUT components is similar to a web network that has a bow-tie structure [10]. However, the production network is different. Ties among the firms form a much tighter network with an overall structure that does not resemble a bow-tie. Then, we study the community structure and reveal its hierarchical nature using the Infomap method [11, 12].

In previous studies [6, 8], the modularity maximization technique [13] is used to study the community structure of the Japanese production network. However, modularity maximization cannot capture the dynamic aspects of the network. This technique reveals a similar type of community partition for both directed and undirected versions of the network. Moreover, it is well known that the modularity maximization algorithm suffers from a resolution limit problem when trying to identify the communities in a large scale network. The map equation method [11, 12] detects communities using the dynamic behaviour of the network. In a recent study [9], the hierarchical map equation is applied to characterize the level 1 communities in the Japanese production network, and a detailed investigation of the topological properties of both the intra and inter communities is conducted. It also shows that the regions and sectors are segregated within the communities. In another study [14], the business cycle correlations of the communities detected by the map equation are studied for the network of firms listed on the Tokyo Stock Exchange. The presence of strong correlations in intra and inter communities is explained by the attributes of both the network topology and the firms. The crucial difference between our paper and [9, 14] is that we not only study the top level communities but also study the communities at the other levels as well as the hierarchical structure. Moreover, we determine the compositions of the communities and subcommunities in terms of whether they include upstream and downstream firms, which has not been investigated in previous studies.

In our paper, we conduct a level-by-level analysis and identify both communities and “irreducible” communities (communities that are not decomposed into subcommunities at the lower level). We also study the overexpression of some of the major communities to identify both the industrial sector and the regional decomposition. The complex nature of the links that exist between the communities are also studied. A discussion and the conclusion as well as suggestions for future research are provided at the end. Some of the supporting materials are included as Appendices.

## Production network data and its basic structure

Our data for the production network are based on a survey conducted by Tokyo Shoko Research (TSR), one of the leading credit research agencies in Tokyo, and was supplied to us through the Research Institute of Economy, Trade and Industry (RIETI). The data were collected by TSR by means of inquiry from firms who represent the top five suppliers and the top five customers. Although the large firms that have many suppliers and customers submitted replies that are incomplete, these data are supplemented with data on the other side of trade: smaller firms submit replies that include data on large firms, who are important trade partners. By combining all the submissions from both side of trade into one database, large firms are connected to numerous smaller firms, which provides a good approximation of the real complete picture. One might worry because some of the trades last for only a short time and sometimes they only occur once, such as when a firm seeks a good deal for just one particular occasion, and thus cast doubt on the definition of the trade network. The form of data collection used for this study solves this problem: it is most implausible that replies containing data on a one-time trade are included, instead, data on firms that maintain a certain trade frequency are likely to be listed. In this study, we use two datasets: ‘TSR Kigyo Jouhou’ (firm information), which contains basic financial information on more than a million firms, and ‘TSR Kigyo Soukan Jouhou’ (firm correlation information), which includes several million supplier-customer and ownership links and a list of bankruptcies. Both of these datasets were compiled in July 2016. (Some of the earlier studies on the production network include [6–9]).

In this study, *i* → *j* denotes a supplier-customer link, where firm *i* is a supplier for another firm *j*, or equivalently, *j* is a customer of *i*. We extracted only the supplier-customer links for pairs of “active” firms and excluded inactive and failed firms by using an indicator flag for them when we retrieved the basic information. We eliminated self-loops and parallel edges (duplicate links recorded in the data), to create a network of firms (as nodes) and supplier-customer links (as edges). The network has the largest connected component when it is viewed as an undirected graph, which is the giant weakly connected component (GWCC) that includes 1,066,037 nodes (99.3% of all the active firms) and 4,974,802 edges.

This study not only analyzes the network but considers several attributes of each node: the financial information in terms of firm size, which is measured as sales, profit, number of employees and the firm’s growth; the major and minor classifications of industrial sectors, details regarding the firm’s products, the firm’s main banks, the principal shareholders, and miscellaneous other information including geographical location. For the purpose of our study, we focus on two attributes of each firm, namely the industrial sector and the geographical location of the head office.

**Fig 1:**
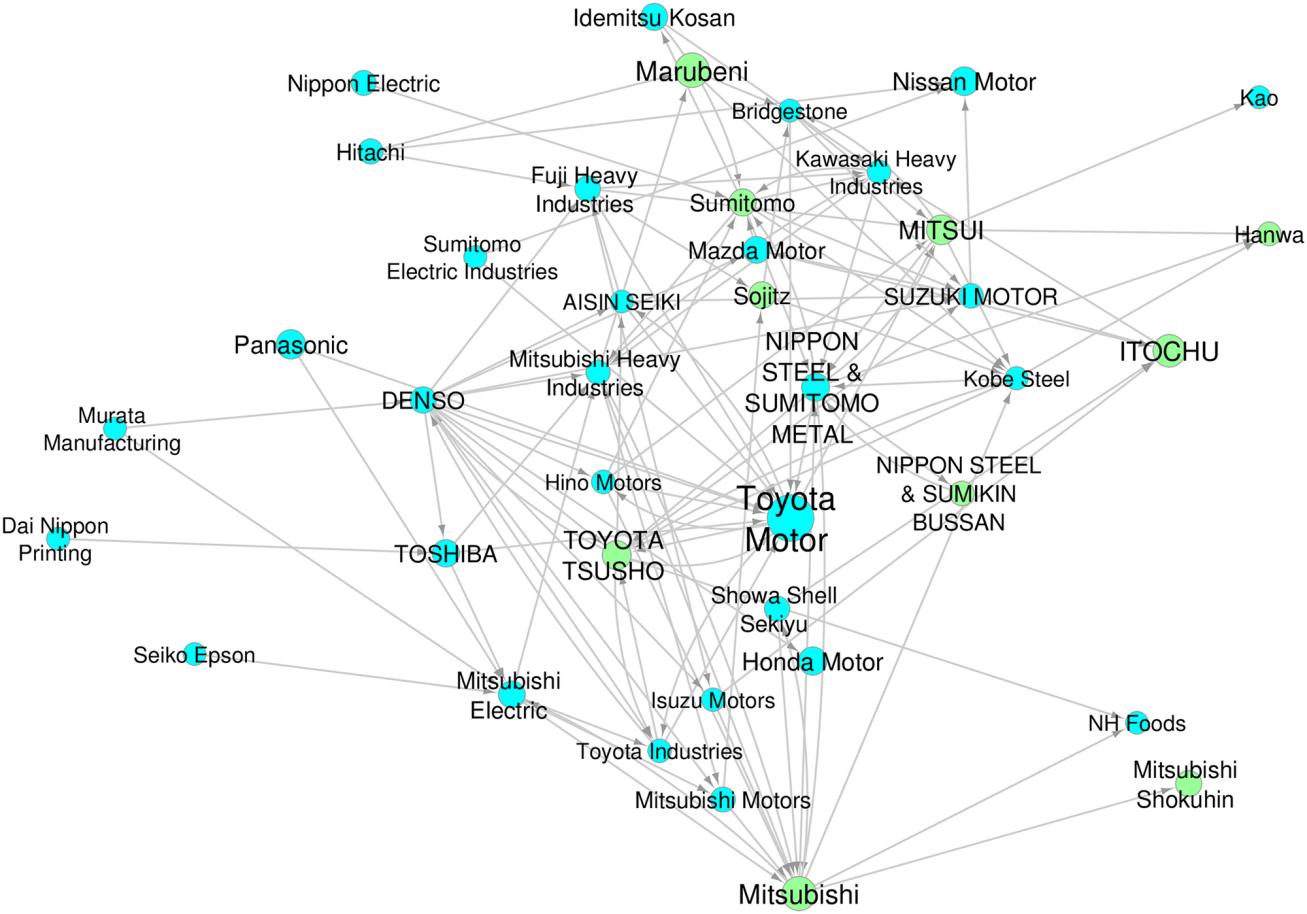
Representative network of the automobile industry in Japan. Major firms are selected under the following conditions: i) they are connected to Toyota Motor within three degrees of separation, ii) they belong to either the manufacturing or wholesale sectors, iii) they are listed in the first section of the Tokyo Stock Exchange, and iv) They are in the top 40 in terms of sales. The firms thus selected are displayed as nodes and the transactions between them are displayed as arrows. All of the displayed nodes belong to the GSCC component. The size of the nodes is scaled to the sales of the corresponding firm. The color of the nodes distinguishes their industry type; blue and green designate manufacturing and wholesale, respectively.

The industrial sectors are hierarchically categorized into 20 divisions, 99 major groups, 529 minor groups and 1,455 industries (Japan Standard Industrial Classification, November 2007, Revision 12). See Table A in S1 Appendix for the number of firms in each division of each industrial sector. Each firm is classified according to the sector it belongs to, and the primary, secondary and tertiary, if any, is identified. The geographical location is converted into a level of one of 47 prefectures or into one of 9 regions (Hokkaido, Tohoku, Kanto, Tokyo, Chubu, Kansai, Chugoku, Shikoku, and Kyushu). See Table B in S1 Appendix for the number of firms in each regional area of Japan. Fig 1 depicts a representative supply-chain network of the automobile industry in Japan. For example, Toyota Motor Corporation, the largest car manufacturer in the nation, obtains mechanical parts from suppliers such as Denso and Aisin Seiki. In addition, Toyota is indirectly connected to Denso through Aisin Seiki. One can also go up from Denso to Murata Manufacturing in the figure. For electronic parts, another important components of cars, Toyota has direct transactions with general electrical manufacturers such as Toshiba and Panasonic, and Toshiba, in turn, obtains parts from Dai Nippon Printing. General trading companies such as Marubeni, Mitsui, and Toyota Tsusho play a key role in the formation of the supply-chain network. In addition, we can observe a circular transaction relation among Toyota Motor, Denso, and Toyota Industries. The existence of such a feedback loop can complicate firms’ dynamics in the production network.

In terms of the flow of goods and services (and money in the reverse direction), the firms are classified in three categories: the “IN” component, the “GSCC”, and the “OUT” component. This structure is called “bow-tie” in a well-known study on the Internet [10]. The GWCC can be decomposed into the parts defined as follows:

**GWCC** the giant weakly connected component: the largest connected component when the network is viewed as an undirected graph. An undirected path exists for each arbitrary pair of firms in the component.
**GSCC** the giant strongly connected component: the largest connected component when the network is viewed as a directed graph. A directed path exists for each arbitrary pair of firms in the component.
**IN** The firms through which the GSCC is reached via a direct path.
**OUT** The firms that are reachable from the GSCC via a direct path.
**TE** “Tendrils”; the remainder of the GWCC

It follows from the definitions that

$$\begin{array}{c} \text{GWCC}=\text{GSCC}+\text{IN}+\text{OUT}+\text{TE} \end{array}$$ (1)

We, however, find it far more appropriate to call this structure a “Walnut” structure, as “IN” and “OUT” components are not as separated as in the two wings of a “bow-tie” but are more like the two halves of a walnut shell, surrounding the central GSCC core. This can be explained as follows. The number of firms in each component of the GSCC, IN, OUT and TE is shown in Table 1. Half of the firms are inside the GSCC. 20% of the firms are in the upstream side or IN, and 26% of them are in the downstream side or OUT.

**Table 1:** Walnut structure: The sizes of the different components.

Component	#firms	Ratio (%)
GSCC	530,174	49.7
IN	219,927	20.6
OUT	278,880	26.2
TE	37,056	3.5
Total	1,066,037	100

“Ratio” refers to the ratio of the number of firms to the total number of the firms in the GWCC.

**Fig 2:**
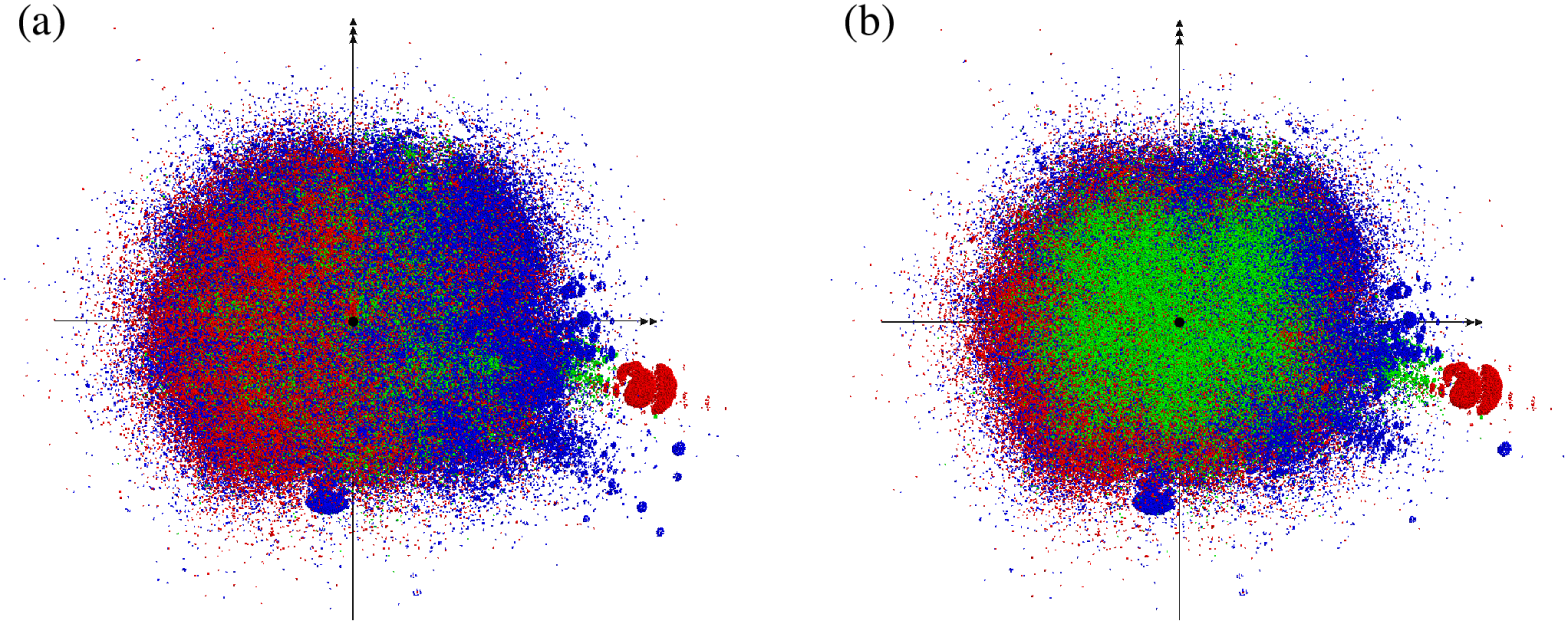
Visualization of the network in three-dimensional space. A surface view of the network is shown in panel (a), and a cross-sectional view that is cut through its center is shown in panel (b). The red, green, and blue dots represent firms in the IN, GSCC, and OUT components, respectively.

In contrast with the well-known “bow-tie structure” in the study conducted by [10] (in which the GSCC is less than one-third of the GWCC), the GSCC in the production network occupies half of the system, meaning that most firms are interconnected by the small geodesic distances or the shortest-path lengths in the economy. In fact, by using a standard graph layout algorithm based on a spring-electrostatic model with three-dimensional space [15], we can show in Fig 2 by visual inspection how closely most firms are interconnected with each other.

**Fig 3:**
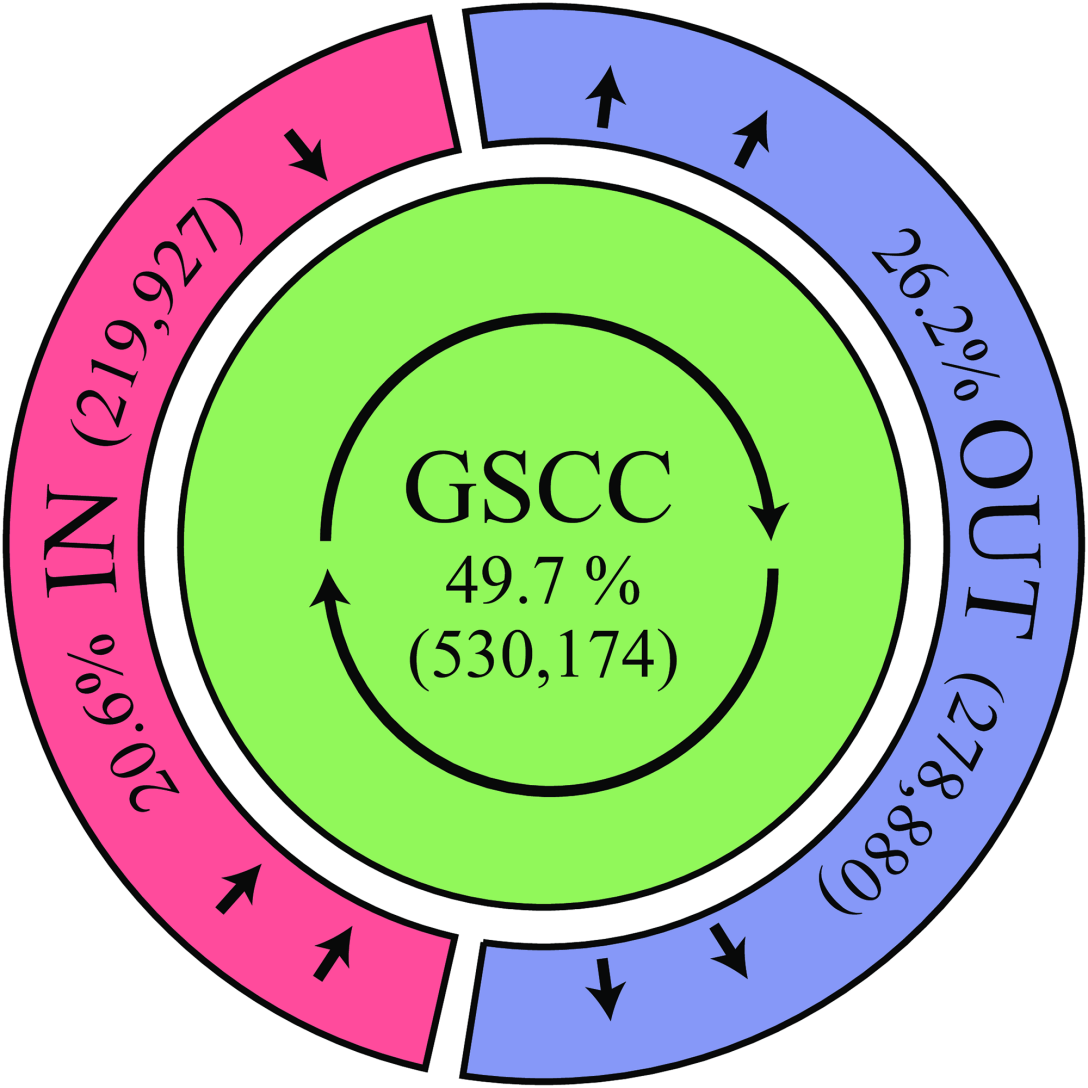
The walnut structure. The production network as a walnut structure. The area of each component is approximately proportional to its size.

Moreover, by examining the shortest-path lengths from GSCC to IN and OUT as shown in Table 2, one can observe that the firms in the upstream or downstream sides are mostly located a single step away from the GSCC. This feature of the economic network is different from the bow-tie structure of many other complex networks. For example, the hyperlinks between web pages of a similar size, (GWCC: 855,802, GSCC: 434,818 (51%), IN: 180,902 (21%), OUT: 165,675 (19%), TE: 74,407 (9%)) which are studied in [16], have a bow-tie structure such that the maximum distance from the GSCC to either IN or OUT is 17, while more than 10% of the web pages in IN or OUT are located more than a single step away from the GSCC. This observation as well as Fig 2 leads us to say that the production network has a “walnut” structure, rather than a bow-tie structure. We depict the schematic diagram in Fig 3.

**Table 2:** Walnut structure: The shortest distance from GSCC to IN/OUT.

IN to GSCC			OUT to GSCC		
Distance	#firms	Ratio (%)	Distance	#firms	Ratio (%)
1	212,958	96.831	1	266,925	95.713
2	6,793	3.089	2	11,650	4.177
3	170	0.077	3	296	0.106
4	6	0.003	4	9	0.003
Total	219,927	100	Total	278,880	100

The left half shows the number of firms in the IN component that connects to the GSCC firms with the shortest distance 1–4. The left side shows the OUT component.

Later, we shall show how each densely connected module or community is located in the walnut structure.

## Methods

### Community detection

Community detection is widely used to elucidate the structural properties of large-scale networks. In general, real networks are highly non-uniform. Community detection singles out groups of nodes densely connected to each other in a network to divide that network into modules. This process enables us to have a coarse-grained view of the structure of such complicated networks. One of the most popular methods used for community division is maximizing the modularity index [13]. Modularity measures the strength of the partition of a network into communities by comparing the fraction of links in given communities with the expected fraction of links if links were randomized with the same degree of distribution as the original network. However, it is well known that the modularity method suffers from a problem called resolution limit [17] when applied to large networks. That is, optimizing modularity fails to detect small communities even if they are well defined, such as cliques.

The map equation method [11] is another method used to detect communities in a network. This method is found to be one of the best performing community detection techniques compared to the others [18]. The map equation method is a flow-based and information-theoretic method depending on the map equation, which is defined as

$$\begin{array}{c} L\left(C\right)=q_{↷}H\left(C\right)+\sum_{i=1}^{m}p_{↻}^{i}H\left(P^{i}\right). \end{array}$$ (2)

Here, *L*(*C*) measures the per step average description length of the dynamics of a random walker migrating through the links between the nodes of a network with a given node partition *C* = {*C*_1_, ⋯, *C_ℓ_*} that consists of two parts. The first term arises from the movements of the random walker across communities, where $q_{↷}$ is the probability that the random walker switches communities, and *H*(*C*) is the average description length of the community index codewords given by the Shannon entropy. The second term arises from the movements of the random walker within the communities, where $p_{↻}^{i}$ is the percentage of the movements within the community *C*_*i*_, and $H(P^{i})$ is the entropy of the codewords in the module codebook *i*.

If the network has densely connected parts in which a random walker stays a long time, one can compress the description length of the random walk dynamics in a network by using a two-level codebook for nodes adapted to such a community structure; this is similar to geographical maps in which different cities recycle the same street names such as “main street’ [11]. Therefore, obtaining the best community decomposition in the map equation framework amounts to searching for the node partition that minimizes the average description length *L*(*C*).

In regard to the resolution limit problem, any two-level community detection algorithms including the map equation are not able to eliminate the limitation. However, the map equation significantly mitigates the problem as has been shown by a recent theoretical analysis [19]. In practice, this is true for our network, as will be demonstrated later.

Recently, the original map equation method has been extended to networks with multi-scale inhomogeneity. A network is decomposed into modules that include their submodules and then their subsubmodules and so forth. The hierarchical map equation [12] recursively searches for such a multilevel solution by minimizing the description length with possible hierarchical partitions. The map equation framework for the community detection of networks is now more powerful. Therefore, we analyze the production network using this method. The code of the hierarchical map equation algorithm is available at http://www.mapequation.org.

Note that this study exclusively considers the community identification for nodes in our network. That is, each node belongs to a unique community at every hierarchical level. However, such community assignment may be too restrictive for a small number of giant conglomerate firms such as Hitachi and Toshiba because of the diversity of their businesses. The map equation is so flexible that it can detect the overlapping community structure of a network in which any node can be a member of multiple communities [20]. However, we use the original algorithm as an initial step toward obtaining a full account of the firm-to-firm transaction data.

### Overexpression within communities and subcommunities

Most real-world networks have a community structure [21]. Such communities are formed in a network based on the principle of homophily [22]. This principle indicates that a node has a tendency to connect with other similar nodes. For example, ethnic and racial segregation are observed in our society [23], biological functions play a key role in the formation of communities in protein-protein interaction networks [24], and the community structure of stock markets is similar to that of their economic sectors [25]. We find that attributes play a crucial role in the formation of the community structure of the production network using the following method.

We follow the procedure used in [26] to determine the statistically significant overexpression of different locations and sectors within a community. This method was developed from the statistical validation of the overexpression of genes in specific terms of the Gene Ontology database [27]. In this procedure, a hypergeometric distribution *H*(*X*|*N*, *N*_*C*_, *N*_*Q*_) is used to measure the probability that *X* randomly selected nodes in community *C* of size *N*_*C*_ will have attribute *Q*. The hypergeometric distribution *H*(*X*|*N*, *N*_*C*_, *N*_*Q*_) can be written as

$$\begin{array}{c} H(X|N,N_{C},N_{Q})=\frac{\left(\begin{array}{c} N_{C}\\ X \end{array}\right)\left(\begin{array}{c} N-N_{C}\\ N_{Q}-X \end{array}\right)}{\left(\begin{array}{c} N\\ N_{Q} \end{array}\right)}, \end{array}$$ (3)

where *N*_*Q*_ is the total number of elements in the system with attribute *Q*. Further, one can associate a *p value* *p*(*N*_*C*,*Q*_) with *N*_*C*,*Q*_ nodes, having attribute *Q* in community *C* with *H*(*X*|*N*, *N*_*C*_, *N*_*Q*_) by the following relation:

$$\begin{array}{c} p\left(N_{C,Q}\right)=1-\sum_{X=0}^{N_{C,Q}-1}H\left(X|N,N_{C},N_{Q}\right). \end{array}$$ (4)

The attribute *Q* is overexpressed within community *C* if *p*(*N*_*C*,*Q*_) is found to be lower than some threshold value *p*_*c*_. As we use a multiple-hypothesis test, we need to choose *p*_*c*_ appropriately to exclude false positives. We assume that *p*_*c*_ = 0.01/*N*_*A*_, as specified in [26], which includes a Bonferroni correction [28]. Here, *N*_*A*_ represents the total number of different attributes (In our study we have *N*_*A*_ = 9 regional attributes) for all the nodes of the system.

## Results

### Hierarchy of communities

**Fig 4:**
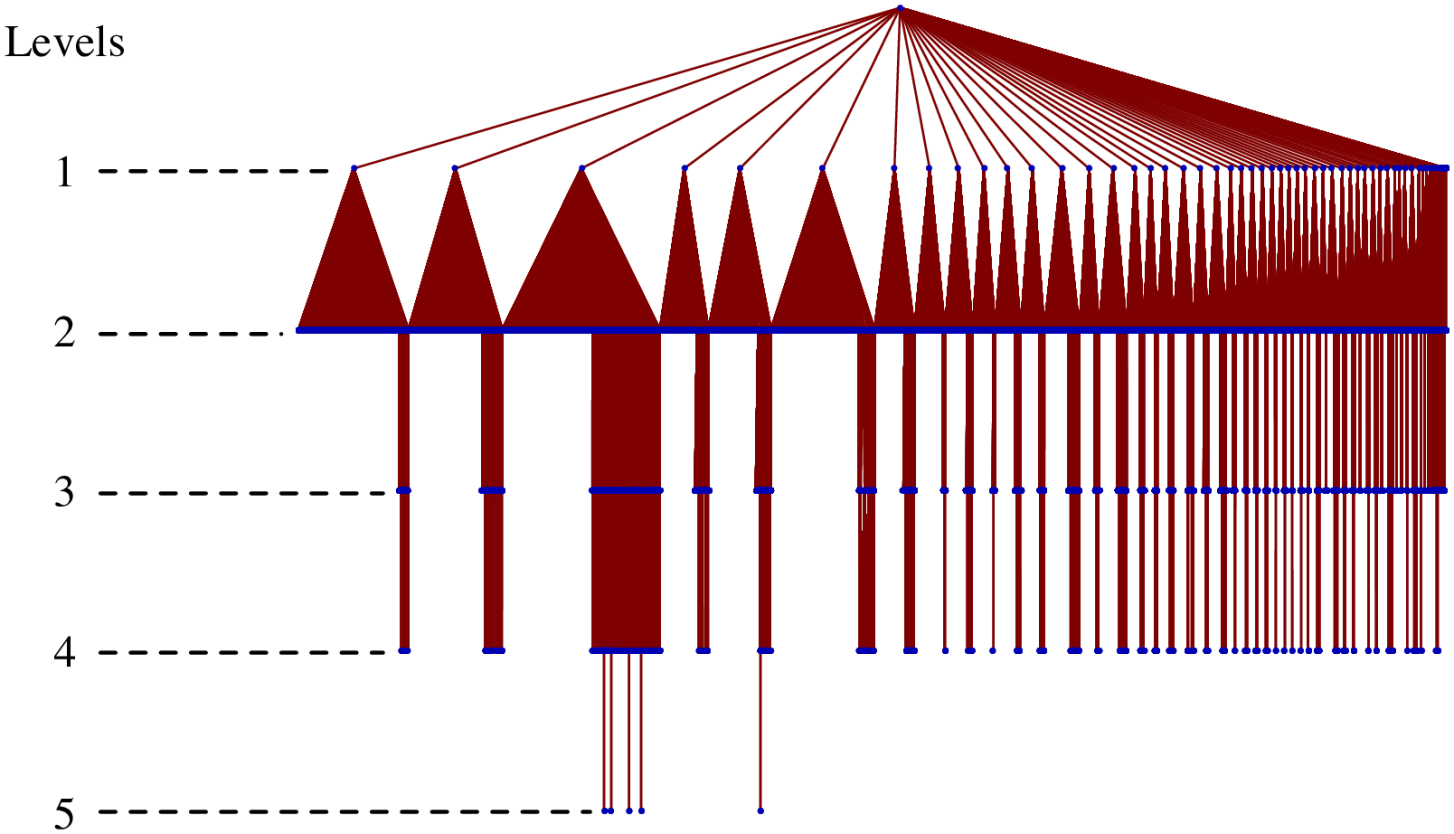
Hierarchical structure of the communities. Five levels of hierarchical community decomposition are illustrated. The width of the triangle originating in each community at the *n*-th level is proportional to the number of its subcomunities at the (*n* + 1)-th level.

By using the Infomap method [11, 12], we find that the communities have a hierarchical structure, as summarized in Table 3, and determine the number of firms at each level. This hierarchical structure is illustrated in Fig 4, where 2nd level communities are lined up from left to right in a descending order in terms of community size (number of firms), and the width of the triangles reflects the number of subcommunities in each community. We find that most of the subcommunites are on the 2nd level and that most of the firms (94%) belong to 2nd level communities. Compared with 1st and 2nd level communities, the 3rd to the 5th levels are of no significant importance. Therefore, we limit our discussion of the properties of the (sub)communities to those of the 2nd level. Past studies on the application of the hierarchical map equation to real world networks [12, 19] show that dense networks have large communities at the finest level with shallow hierarchies, and sparse networks tend to have deep hierarchies. It is also observed that the depth of the hierarchies increases with network size. In the case of the California road network, the hierarchy has a deep level because the road network has geographical constraints that decrease the number of shortcuts between the different parts of the network [12]. In our production network, we observe a relatively shallow hierarchy because it does not have such strict constraints.

**Table 3:** Modular level statistics.

Level	#com	#irr.com	#firms	Ratio (%)
1	209	106	830	0.078
2	65, 303	60, 603	998,267	93.643
3	18, 271	17, 834	61,748	5.792
4	1, 544	1,539	5,168	0.485
5	10	10	24	0.002
Total		80,092	1,066,037	100.00

Results of community detection using the multi-coding Infomap method. “#com” is the number of all the communities, “#irr.com” is the number of irreducible communities, which are communities that do not have any subcommunities. “#firms” refers to the number of firms in irreducible communities

**Fig 5:**
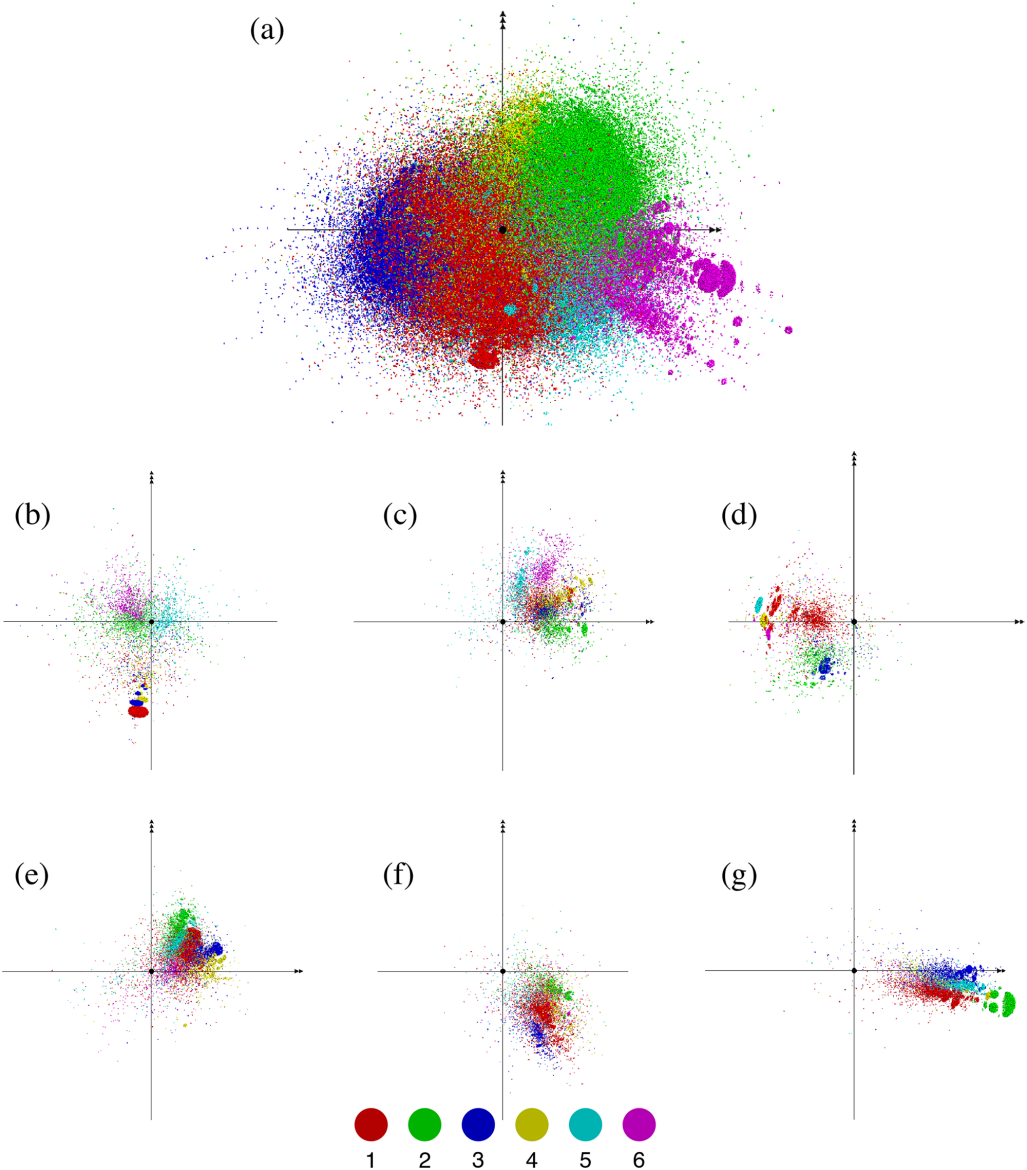
Hierarchical decomposition of the whole network into communities and subcommunities. This panel (a) highlights the 6 largest communities at the top modular level with different colors. Each of these communities is further decomposed into subcommunities as demonstrated in panels (b) through (g), where the 6th largest subcommunities of the 1st through the 6th largest communities are highlighted.

We visualize the hierarchical decomposition of the whole network into communities and their subcommunities in Fig 5. The configuration of the nodes in three-dimensional space is the same as that in Fig 2. We can see that the network is extremely complex with multi-scale inhomogeneity. The results of an overexpression analysis indicate that the major communities of the 1st and 2nd levels are characterized as industrial sectors and regions, as noted in the subsequent subsections.

For the purpose of making the following discussion of communities transparent, let us adopt the following indexing convention: At the top modular level of the hierarchical tree structure, the communities are indexed by their rank in size (the number of firms in the community). Thus, the largest community at the top level is denoted as “C_1_”. At the lower levels, the rank of the size is added after ‘:’. For example, community “C_1:5_” is the fifth largest 2nd level community among all the 2nd-level communities that belong to the largest top-level community C_1_.

### Level-1 communities

**Fig 6:**
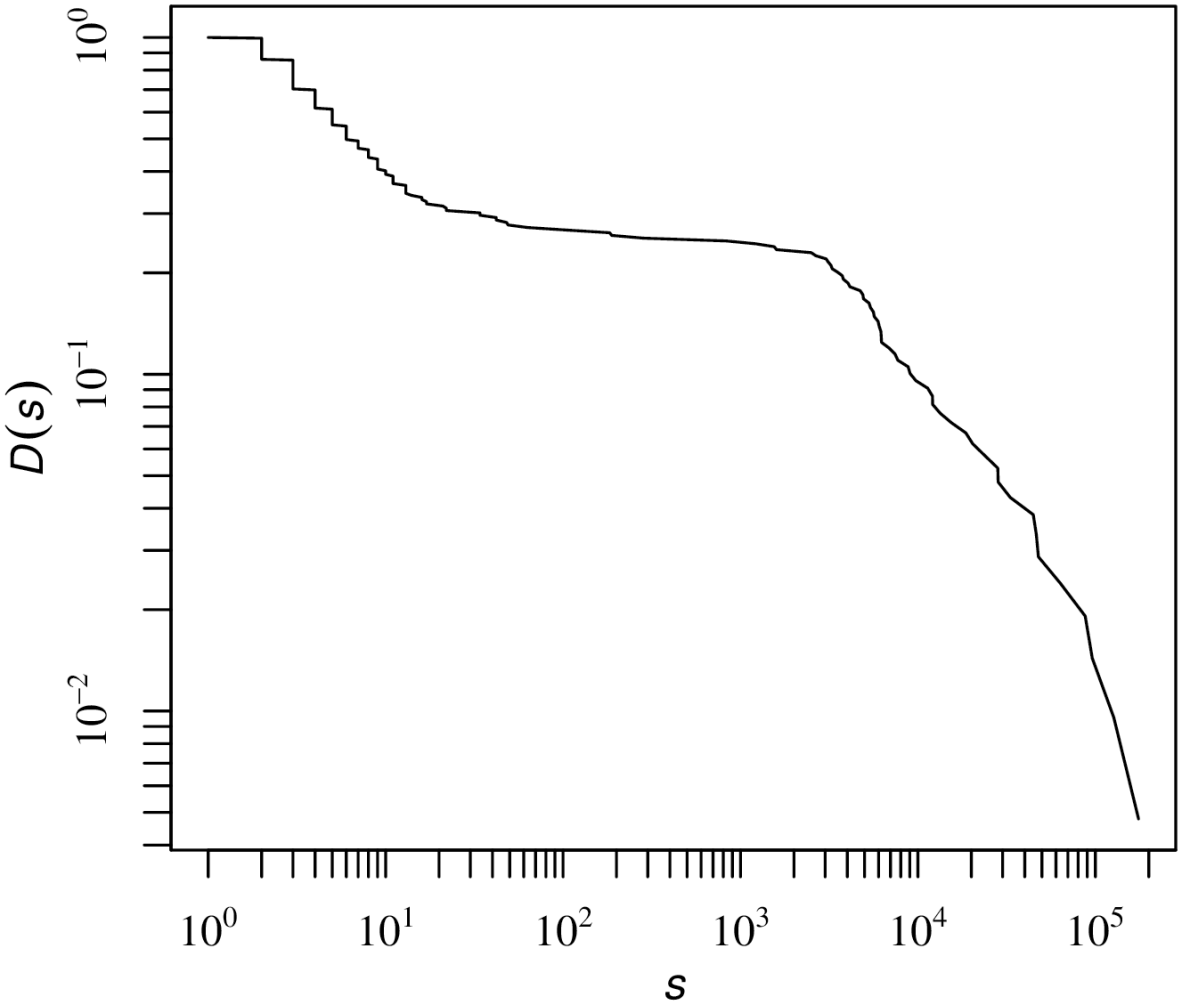
The complementary cumulative distribution function *D*(*s*) of the community size *s* at the top modular level.

The complementary cumulative function *D(s)* indicates the fraction of communities at the top level having a size of at least s, as shown in Fig 6. The bimodal nature of the distributions manifests the resolution limit problem. A small number of communities predominates the whole system. Among some 200 communities detected, for example, the largest communities contain 100,000-200,000 firms. However, such extremely large communities are decomposed into subcommunities by the hierarchical map equation in a unified way. This process is quite different from community detection based on modularity. One may address this problem by applying the modularity maximization method recursively; communities are regarded as separated subnetworks that can be further decomposed. However, this procedure lacks a sound basis because it uses different null models to decompose the subnetworks [21]. A more detailed comparison between these two methods is provided in S1 Appendix.

**Fig 7:**
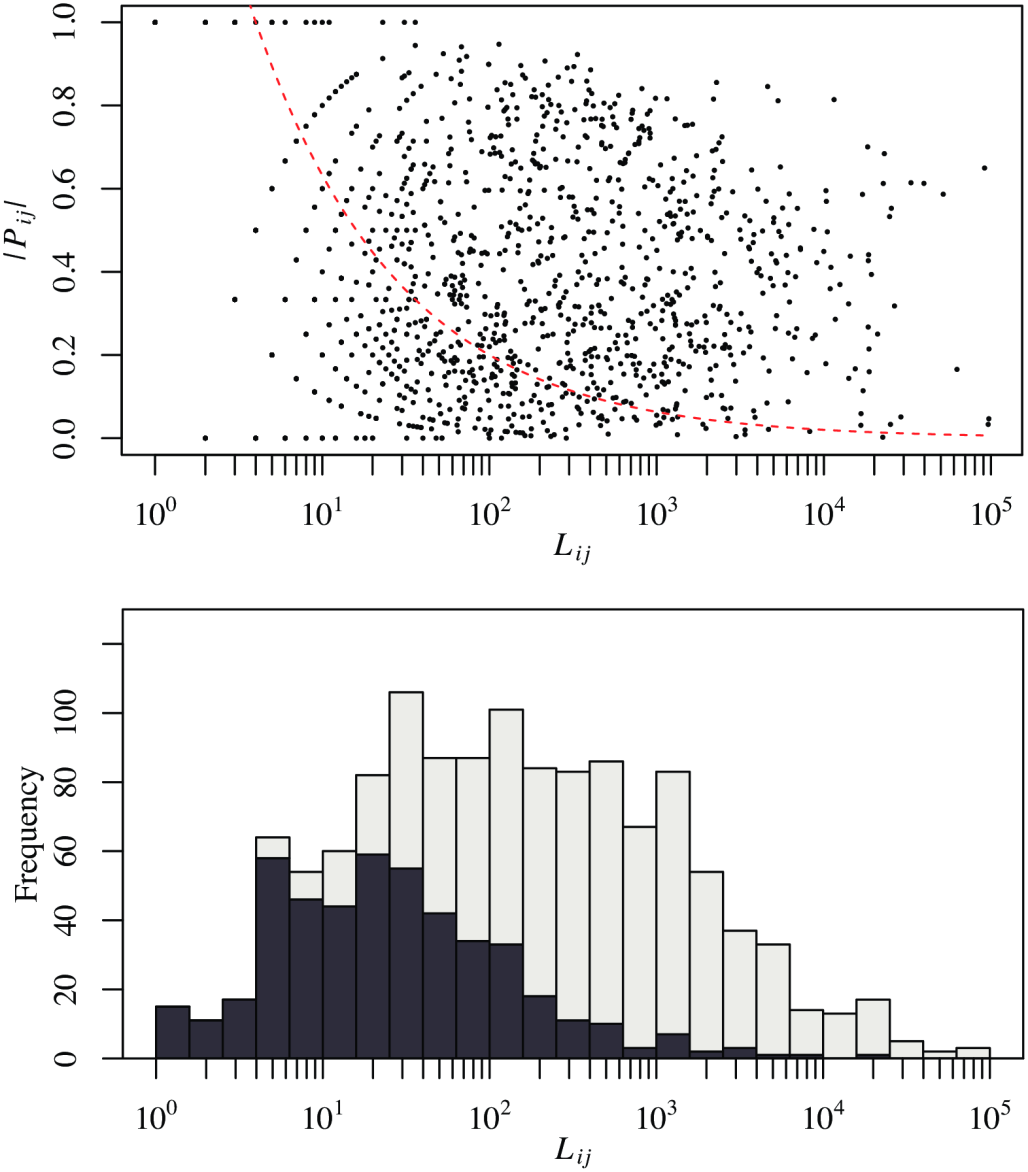
Polarizability of the direction of links interconnecting communities at the top level. Here, 51 major communities containing more than 1,000 firms are selected. The top figure plots the polarization ratio |*P*_*ij*_| of the linkage between communities *i* and *j* versus the total number *L*_*ij*_ of its constituting links. The dashed curve shows the significance level corresponding to 2*σ* for the polarizability of intercommunity linkage for the given total number of its constituents, where the random orientation of the individual links is adopted as a null model; see Eq (6) for the standard deviation *σ*. The bottom figure is a histogram for the frequency of intercommunity linkages in each bin of *L*_*ij*_. The grey (black) bars depict the number of intercommunity linkages with a |*P*_*ij*_| that is higher (lower) than the threshold for the test of statistical significance.

The map equation is a method that can be used to divide a directed network into communities in which nodes are tightly connected in both directions. Due to the nature of the network, the flows across communities thus detected should be biased in an either direction. Fig 7 confirms this expectation. To quantify the polarizability of the links between a pair of communities, we introduce the polarization ratio defined by

$$\begin{array}{c} P_{ij}=\frac{A_{ij}-A_{ji}}{A_{ij}+A_{ji}}, \end{array}$$ (5)

where *A*_*ij*_ is the total number of links spanning from communities *i* to *j* and *A*_*ji*_ and that of the opposite links. If the linkage between communities *i* and *j* is completely polarized, then *P*_*ij*_ becomes ±1 depending on its direction; if the linkage is evenly balanced, then *P*_*ij*_ = 0. If we assume that the links have no preference with respect to their direction as a null hypothesis, then the null model predicts that the polarization ratio for the connections between communities *i* and *j* fluctuates around 0 with the standard deviation *σ* given by

$$\begin{array}{c} \sigma =\frac{1}{\sqrt{L_{ij}}}\;, \end{array}$$ (6)

where *L*_*ij*_ = *A*_*ij*_ + *A*_*ji*_ is the total number of links between the two communities. If we focus on intercommunity linkages with *L*_*ij*_ ≥ 100, we see that the ones whose direction is polarized in a statistically meaningful way occupy 86.7% of their total. The corresponding share of intercommunity linkages is 70.1% for *L*_*ij*_ ≥ 10. Most of the connections between communities with more than 100 links are significantly polarized in reference to the random orientation model for intercommunity links.

We find the overexpression of the attributes in 1st level communities to determine the factors that play a crucial role in the formation of such communities. Our study considers both the location and the sector attributes. The location attributes are divided into 9 regions, and the sector attributes are categorized in 20 divisions. The details about the sixth largest 1st level communities and the overexpressed attributes within it are tabulated in Table 4. We also use a finer classification, i.e., 47 prefectures and 99 major sectors for which the results are provided in S1 Appendix. We observe a strong connection between overexpressed sectors and overexpressed regions. In the largest community, mainly manufacturing sectors and heavily urbanized regions (Kanto, Tokyo, Chubu, and Kansai) are overexpressed. The 2nd largest community shows that mainly the agriculture and food industries (see SI) and rural regions (Hokkaido, Tohoku, Shikoku, and Kyusyu-Okinawa) are overexpressed. In terms of overexpression in the 3rd largest community, the construction sector dominates and the corresponding overexpressed region indicates these firms are mainly based in Kanto and Tokyo. The transport and wholesale retail trade industries are the dominate attributes of the 4th largest community, and Tohoku, Kanto, and Chubu are the overexpressed regions. The 5th largest community mainly includes Tokyo, and the primary overexpressed sectors are information and communications, scientific research, and professional and technical services. The 6th largest community primarily primarily includes medicine and health care. To summarize, the following characterizes the six largest communities:

- The largest community: Manufacturing sectors
- The second largest community: Food sectors
- The third largest community: Construction sectors
- The fourth largest community: Wholesale and retail trade
- The fifth largest community: IT sector and scientific research, primarily based in Tokyo
- The sixth largest community: Medical and health care

**Table 4:** Overexpressions of the 1st level communities.

Index	Size	#subcom	Region	Sector	IN	GSCC	OUT
1	175,150	7135	Kanto (0.21); Tokyo (0.14); Chubu (0.22); Kansai (0.21)	Manufacturing (0.33);	0.20	0.65	0.14
2	126,997	5455	Hokkaido (0.07); Tohoku (0.11); Shikoku (0.05); Kyusyu-Okinawa (0.13)	Agriculture (0.04); Manufacturing (0.18); Wholesale and retail (0.43); Accommodations (0.11); Living-related (0.03); Compound services (0.02)	0.11	0.46	0.40
3	96,062	7339	Kanto (0.48); Tokyo (0.25)	Construction (0.64); Real estate (0.09); Scientific research (0.06);	0.39	0.38	0.16
4	87,647	2660	Tohoku (0.11); Kanto (0.22); Chubu (0.20)	Transport (0.15); Retail (0.38); Finance (0.05); Services, N.E.C. (0.17)	0.11	0.43	0.44
5	63,611	3631	Tokyo (0.40)	Information (0.25); Finance (0.01); Real estate (0.05); Scientific research (0.13); Living-related (0.05); Education (0.01); Services, N.E.C. (0.07)	0.26	0.45	0.26
6	47, 759	6214	Hokkaido (0.06); Tokyo (0.22); Chugoku (0.08); Shikoku (0.05); Kyusyu-Okinawa (0.13)	Wholesale and retail (0.28); Living-related (0.05); Medical (0.48)	0.24	0.21	0.52

“#subcom” is the total number of subcommunities included in each of the 1st level communities. The overexpression in terms of the regions and sector-divisions of the 6th largest communities at the 1st level. The percentage of nodes having a particular attribute is indicated in parentheses. Those with less than 0.01 are not listed. In addition, the percentages of the IN, GSCC, and OUT components are listed for each community.

**Fig 8:**
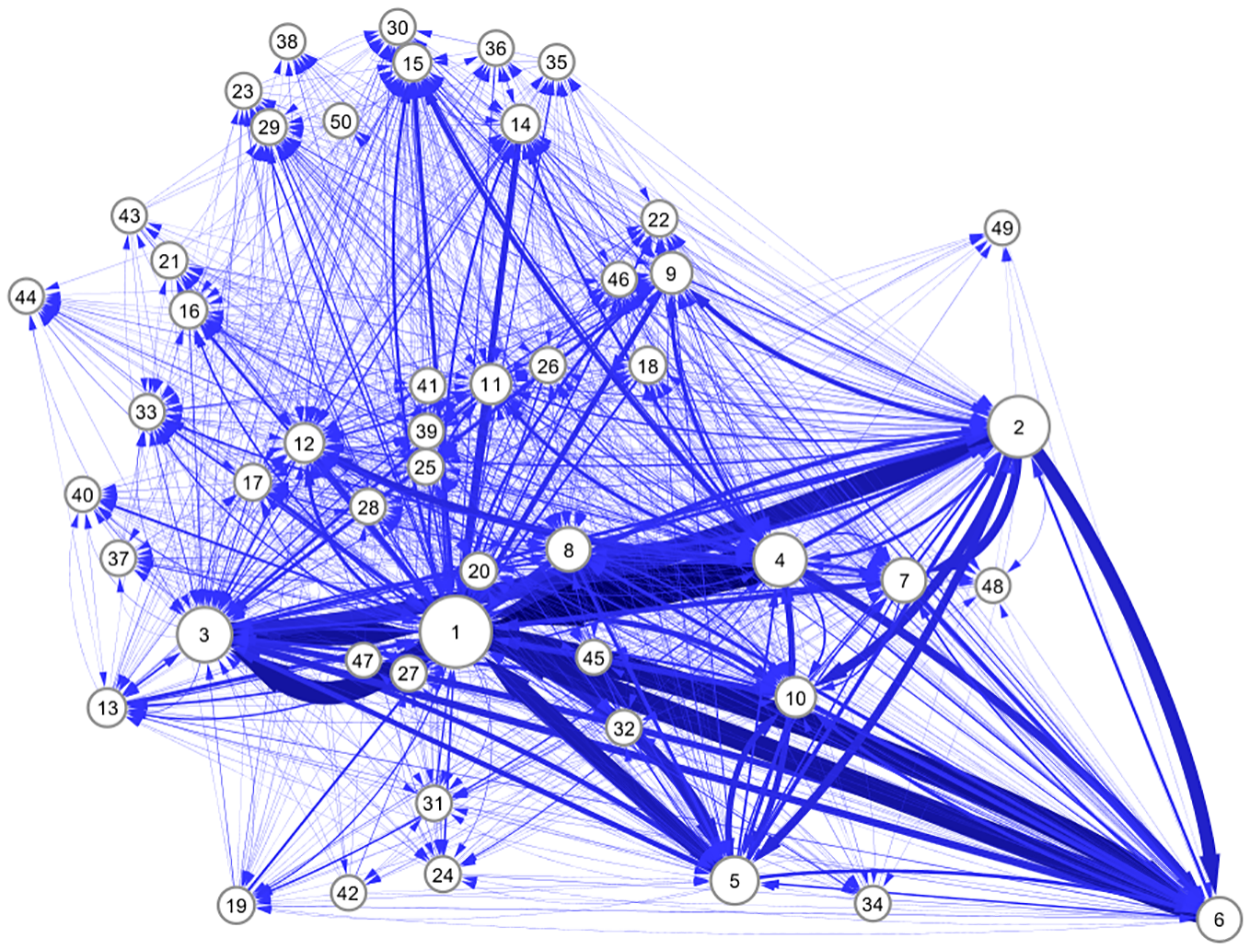
Network of the 50 largest communities at the top level. The major communities are depicted as nodes, and their size is scaled to the size of their corresponding communities. A bundle of directed links connecting a pair of nodes in either direction is represented by an arrow, the width of which is proportional to the total number of their links.

Fig 8 is a coarse-grained diagram of the network shown in Fig 2, where the 50 largest communities at the top level are represented by nodes, and the direct links connecting them, in either direction, are bundled into arrows. We used the following steps to prepare the diagram. We first calculated the center of mass for the IN, GSCC, and OUT components in three-dimensional space. The three centers thus obtained determine the two-dimensional plane for the drawing. Second, we fixed the horizontal axis to optimally represent the direction of flow from the IN (left-hand side) components to the OUT (right-hand side) components through the GSCC; in fact, the three centers are almost aligned horizontally. Then, we calculated the center of mass of the major communities and projected them onto the two-dimensional plane to layout the major communities onto it. Finally, we connected these communities by arrows using information on the links between them.

The positions of the communities on the horizontal line clearly reflect their characteristics in terms of the walnut structure, as shown in Table 4. Among the 6 largest communities, the 3rd community contains twice as many IN components as the averaged concentration on the leftmost side. On the other hand, the 6th community with the largest OUT concentration is on the rightmost side. The 2nd and 4th communities, which are dominated by OUT components, are also on the right-hand side. The 1st community with excess GSCC components is between the 3rd community and the OUT-excess communities. The 5th community, whose composition is very close to the average one, is rather in middle of the walnut structure. Most of the remaining relatively small communities are localized on the left-hand side. This configuration is understandable, because the IN and GSCC components tend to form integrated communities, as will be shown later.

### Level-2 communities

At the 2nd level, some of the top level communities are decomposed to several subcommunities as shown in Tables D and E in S1 Appendix.

**Fig 9:**
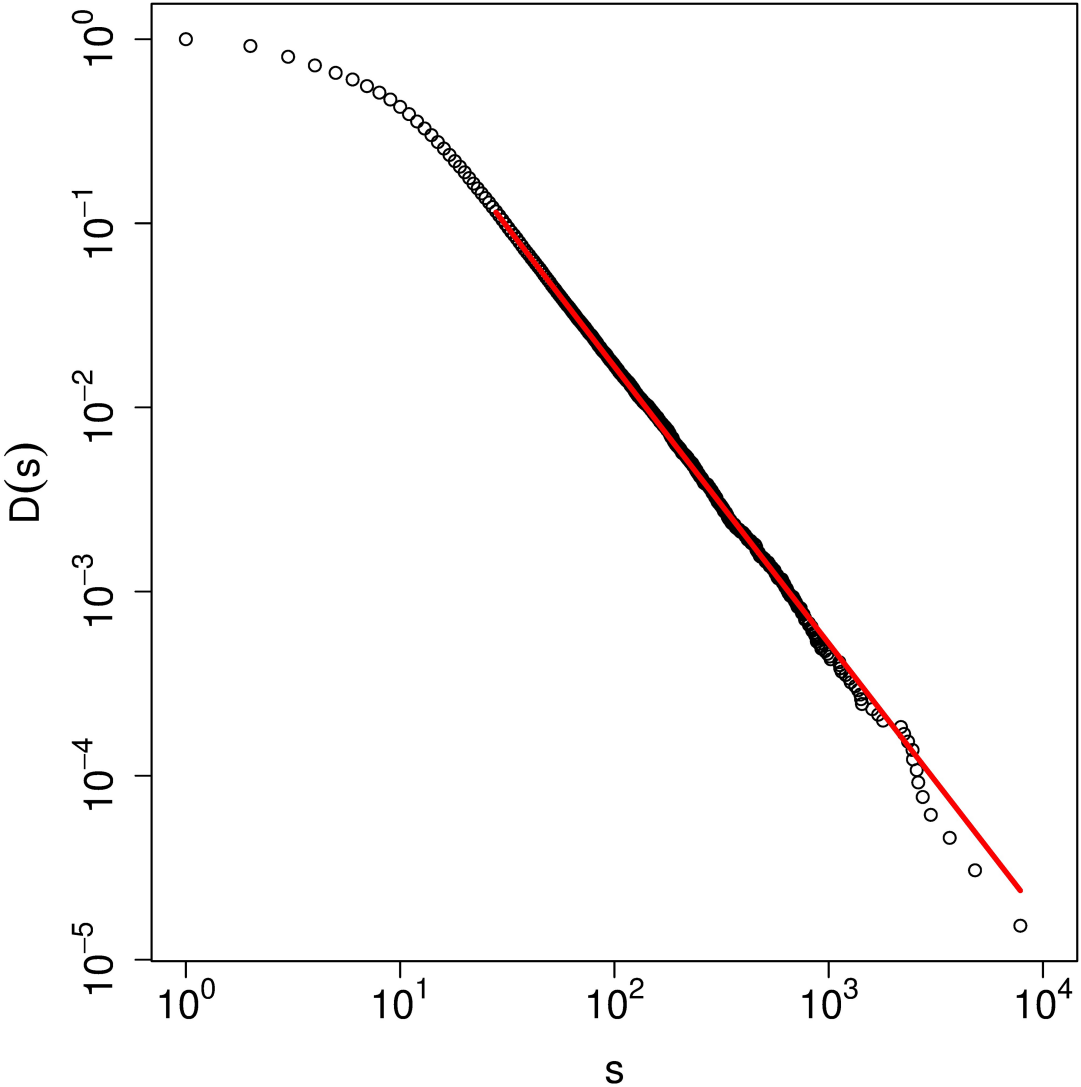
(color online) The complementary cumulative distribution function *D*(*s*) of a community with size *s* at the second modular level. A power-law fit to the data (red line) using the maximum likelihood estimation technique yields *D*(*s*) ∼ *s*^−*γ*+1^ with *γ* = 2.50 ± 0.02, *s*_*min*_ = 28.2 ± 7.6, and *p* *value* = 0.976.

The cumulative distribution of the community size at this level is plotted in Fig 9. We use maximum likelihood estimation (MLE) [29] to quantitatively fit a statistically significant power-law decay for the tail of the CCDF, which has the functional form *D*(*s*) ∼ *s*^−*γ*+1^ with *γ* = 2.50 ± 0.02. The results indicate that the size of the communities is highly heterogeneous and spans over several orders of magnitude.

We also analyzed the overexpressions of selected subcommunities. In terms of subcommunities, we observe wholesale and retail trade is the dominate overexpress attribute of the five largest subcommunities of the largest community. The Kansai region is the only overexpressed region in the 2nd largest subcommunity of the largest community. In *C*_2:1_, transport and postal activities, accommodations, eating and drinking services, living related and personal services, and amusement services dominate the overexpressed sectors, which are mainly based in urban regions (Tokyo and Chubu). The manufacturing, wholesale and retail trades in Tokyo and the Kansai region are overexpressed in *C*_2:2_. Wholesale and retail trade dominate the overexpressed attribute in *C*_2:3_, *C*_2:4_ and *C*_2:5_. A detailed account of the results is provided in S1 Appendix.

**Fig 10:**
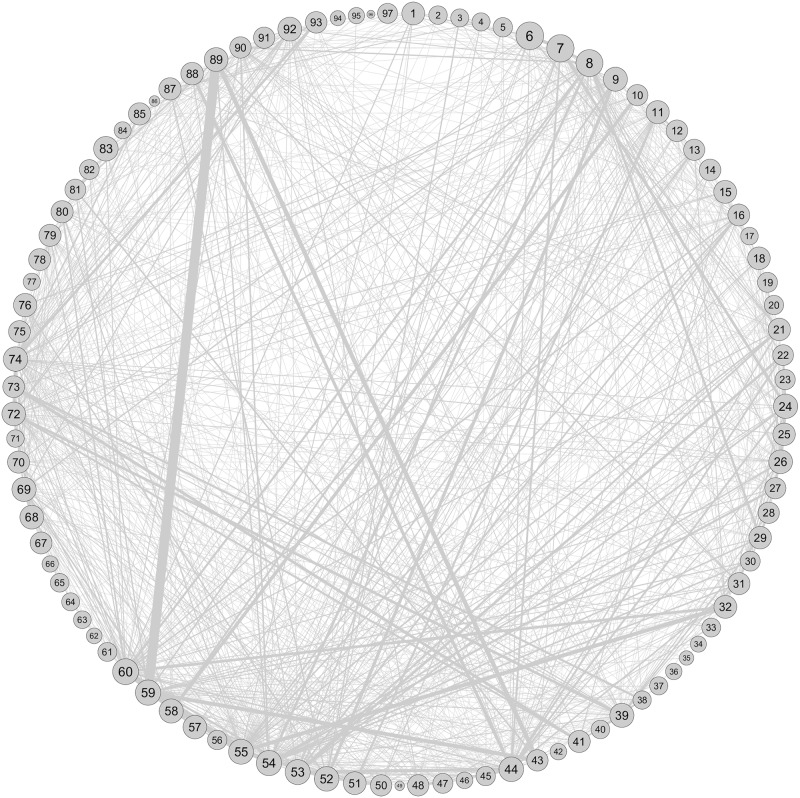
Overexpression network of sectors. The node size represents the percentage of firms belong to that particular sector.

**Fig 11:**
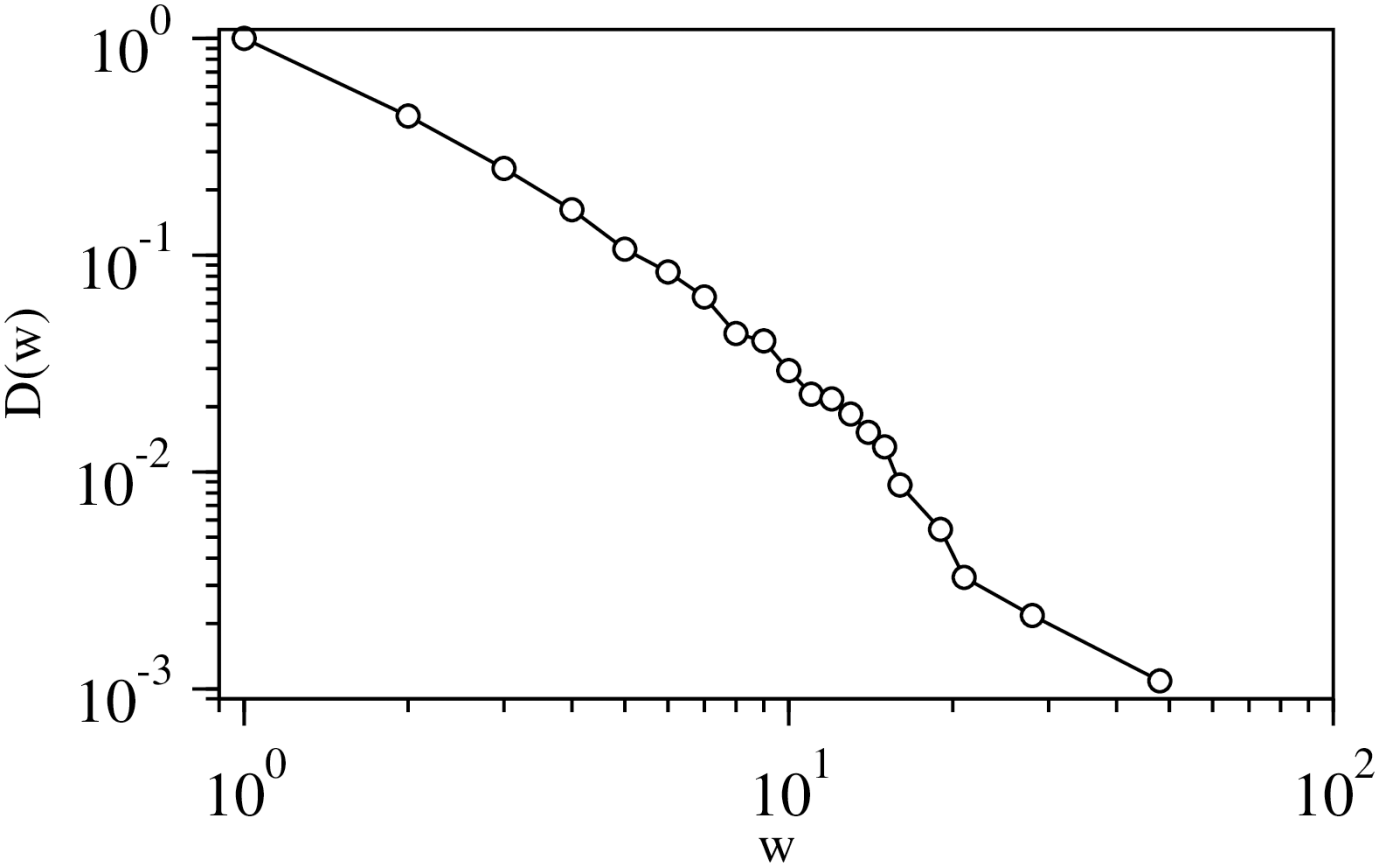
The complementary cumulative distribution of link-weight in the overexpression network.

The network diagram in Fig 10 shows the overlapping nature of the industrial sectors in the communities. We construct a weighted undirected network of 97 major sectors from sector over expression data for the 2nd modular level. Here, a weighted link of value 1 is formed between a pair of sectors if they are overexpressed in the same community. The link-weight of the network is found to be highly heterogeneous with a horizontal distribution as shown in Fig 11. The top five heaviest weighted links between the sectors are listed in Table 5.

**Table 5:** Top five heaviest weighted links between sectors.

Rank	Node 1	Node 2	Weight
1	Retail trade (machinery and equipment)	Automobile maintenance services	48
2	Miscellaneous wholesale trade	Miscellaneous retail trade	28
3	Road passenger transport	Automobile maintenance services	21
4	Miscellaneous manufacturing industries	Miscellaneous wholesale trade	19
5	Road passenger transport	Retail trade (machinery and equipment)	19

**Fig 12:**
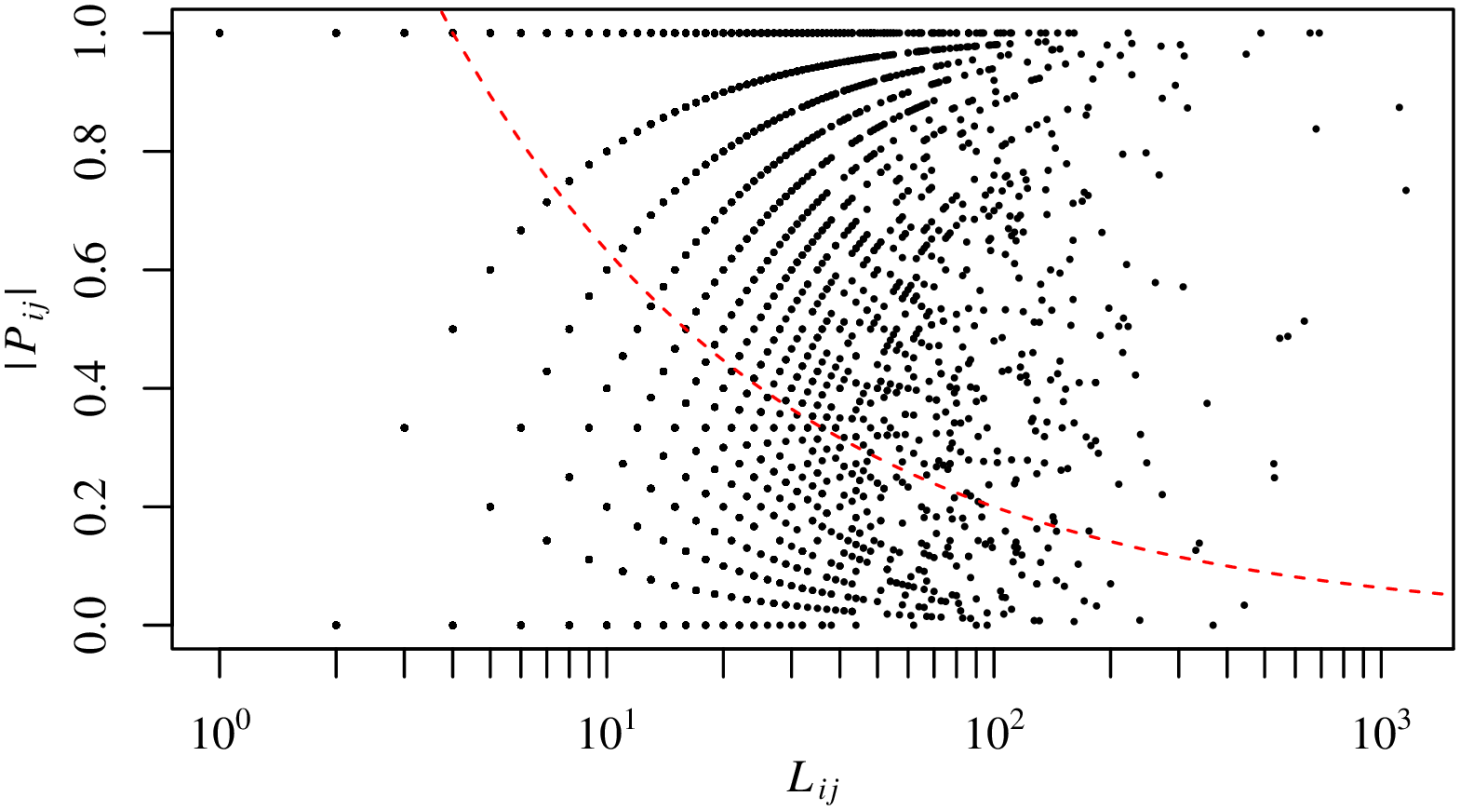
Polarizability of the direction of the links interconnecting communities at the second level. Here, 1086 communities containing over 100 firms are selected. The dashed curve represents the same significance level as in Fig 7.

Fig 12 is the same plot as Fig 7, but this new plot includes communities at the 2nd modular level. We can confirm that the links between the subcommunities are well polarized. Once again, this result is consistent with the nature of the map equation, which extracts communities of tightly connected nodes in a bidirectional way in a directed network.

**Fig 13:**
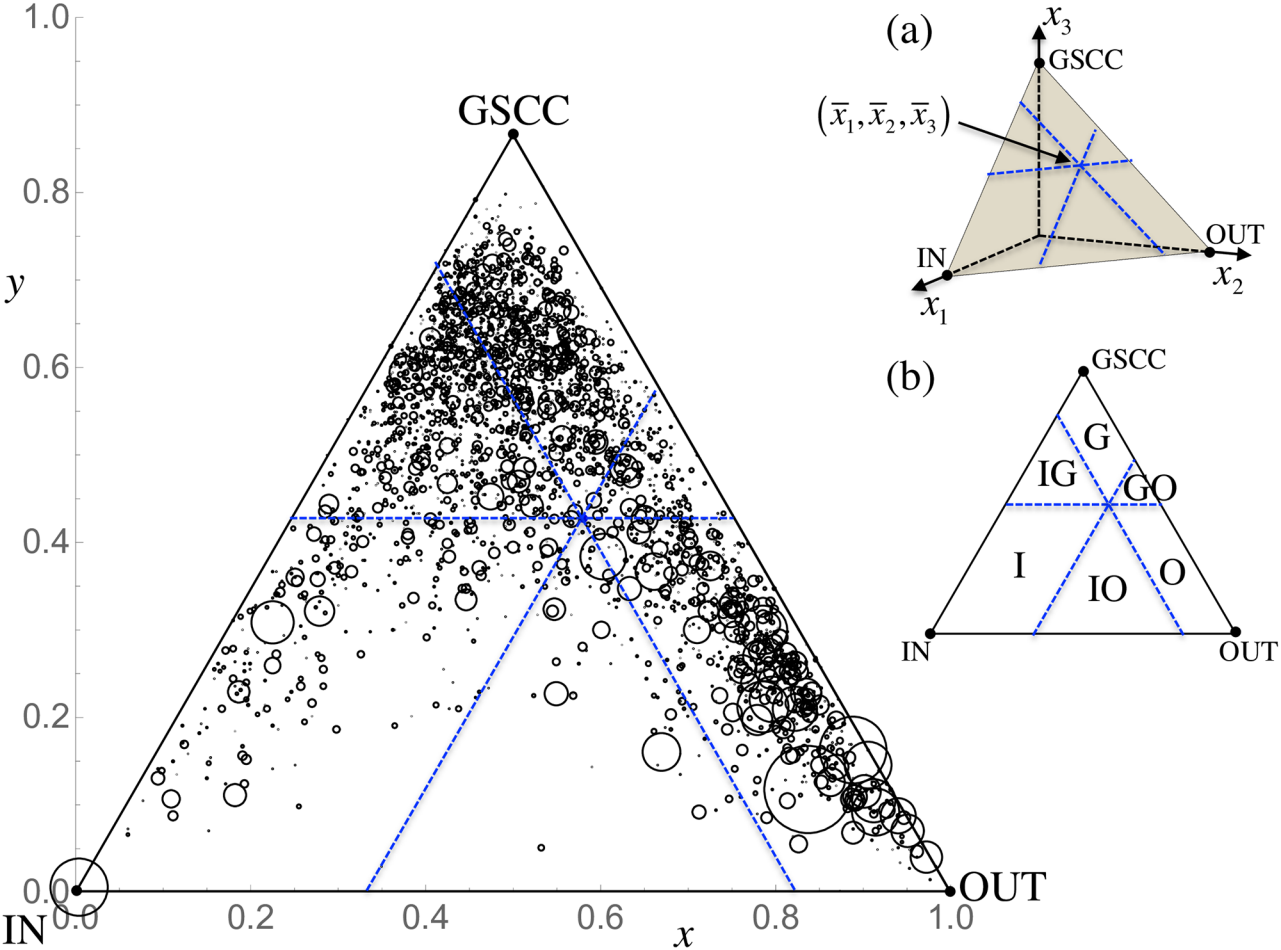
Triangular diagram classifying communities at the second level by their relationship with the walnut structure. Each community is depicted by a circle located at point (*x*, *y*) inside the equilateral triangle, which corresponds to the composition (*x*_1_, *x*_2_, and *x*_3_) of firms belonging to the IN, OUT, and GSCC components that are represented in three-dimensional space; the one-to-one correspondence between (*x*, *y*) and (*x*_1_, *x*_2_, *x*_3_) is illustrated in the associated figure (a). The size of the communities is reflected by the area of their associated circles. The triangular region is decomposed into six domains with the average composition (${\bar{x}}_{1}$, ${\bar{x}}_{2}$, ${\bar{x}}_{3}$) of the IN, OUT, and GSCC components for all firms, as designated in the associated figure (b); see the text for more detailed information on the domain decomposition.

Fig 13 shows how mixed the IN, OUT, and GSCC components of the walnut structure are in each of the large communities with more than 50 firms at the 2nd level, adopting a triangular diagram representation. We exclude firms belonging to TE; however, these are minor components of the walnut structure. Here, 3,011 communities containing more than 50 firms are selected, for a total of 421,779 firms. Suppose that a community contains firms belonging to the IN, OUT, and GSCC components for which the percentages are given by *x*_1_, *x*_2_, and *x*_3_, respectively. The walnut composition of the community is described by point (*x*_1_, *x*_2_, *x*_3_) on the plane of *x*_1_ + *x*_2_ + *x*_3_ = 1 in three-dimensional space. One can thereby establish one-to-one correspondence between a point inside an equilateral triangle and a composition of the three walnut components. The averaged composition of all the firms in the selected communities (i.e., the total number of firms in the IN/OUT/GSCC components divided by the total number of firms in the selected communities) is given by ${\bar{x}}_{1}=0.174$, ${\bar{x}}_{2}=0.333$, and ${\bar{x}}_{3}=0.493$. The triangular region in Fig 13 is then decomposed into six domains in reference to ${\bar{x}}_{1}$, ${\bar{x}}_{2}$, and ${\bar{x}}_{3}$: the communities in domain G ($x_{1}<{\bar{x}}_{1}$, $x_{2}<{\bar{x}}_{2}$, $x_{3}>{\bar{x}}_{3}$) are GSCC-dominant; those in IG ($x_{1}>{\bar{x}}_{1}$, $x_{2}<{\bar{x}}_{2}$, $x_{3}>{\bar{x}}_{3}$) are GSCC-IN hybrid; those in I ($x_{1}>{\bar{x}}_{1}$, $x_{2}<{\bar{x}}_{2}$, $x_{3}<{\bar{x}}_{3}$) are IN-dominant; those in IO ($x_{1}>{\bar{x}}_{1}$, $x_{2}>{\bar{x}}_{2}$, $x_{3}<{\bar{x}}_{3}$) are IN-OUT hybrids; those in O ($x_{1}<{\bar{x}}_{1}$, $x_{2}>{\bar{x}}_{2}$, $x_{3}<{\bar{x}}_{3}$) are OUT-dominant; and those in GO ($x_{1}<{\bar{x}}_{1}$, $x_{2}>{\bar{x}}_{2}$, $x_{3}>{\bar{x}}_{3}$) are GSCC-OUT hybrids. The total number of communities and firms in each domain are listed in Table 6. We observe that there are relatively fewer communities in the I domain and more communities in the IG domain. The IN components thus tend to combine with the GSCC components to form a single community. On the other hand, there are an appreciable number of communities dominated by the OUT components, leading to relatively few communities of IN-OUT and GSCC-OUT hybrids. This tendency, in terms of the characteristics of the communities, may reflect the industrial structure of Japan, which imports raw materials and produces a wide variety of goods out of these for both export and domestic consumption. We are also interested in what occurs in other countries. Once data on the production networks of other countries is available, we hope to compare their community characteristics with those of Japan.

**Table 6:** Classification of communities at the second level based on the walnut structure.

Domain	#com	#firms
G	1,010	114,399
IG	841	92,163
I	294	44,563
IO	80	14,362
O	640	139,986
GO	146	16,306
Total	3,011	421,779

“#com” and “#firms” refer to the total number of communities and firms, respectively, in each of the six domains defined in Fig 13(b).

Although the IN components tend to to merge with the GSCC, we can see the large circle at the vertex of Fig 13. On the other hand, Table 2 shows that most nodes in the IN component have a distance of 1 from the GSCC. Therefore, one may think that there is a large community almost purely composed of nodes in the IN components of the Walnut shape (Fig 3). Actually, this configuration indicates an interesting structure where the nodes are mutually connected and simultaneously connected to nodes in the GSCC. It can be precisely said that the community is in the shape of a walnut shell.

## Comparison of industrial sectors

As is mentioned in the Introduction Section, detecting communities in the supply-chain network is crucial for understanding the agglomerative behavior of firms. This type of research is important because the detected communities are densely connected, and it is plausible that these firms affect each other through the links.

On the other hand, industrial sectors commonly label firms, and these labels are widely used in the economics literature. If there is no difference between the detected communities and the industrial sectors, then there is no reason to make an effort to detect these communities. Therefore, in this section, we show how the detected communities are different from industrial sectors in terms of the interconnections between the groups.

Although different classifications are used for industrial sectors, we discuss the one used in the input-output table [30]. We use this classification because the input-output table is a major research domain in economics, and, more importantly, the purpose of the input-output table is to discuss money flows, which corresponds to the purpose of this paper.

As previously mentioned, there are 209 communities in the 1st level and 66,133 communities in the 2nd level. On the other hand, the input-output tables have 13, 37, 108, 190, and 397 sectoral classifications, which are nested. We choose to compare 209 communities and 190 industrial sectors because these numbers are comparable.

**Fig 14:**
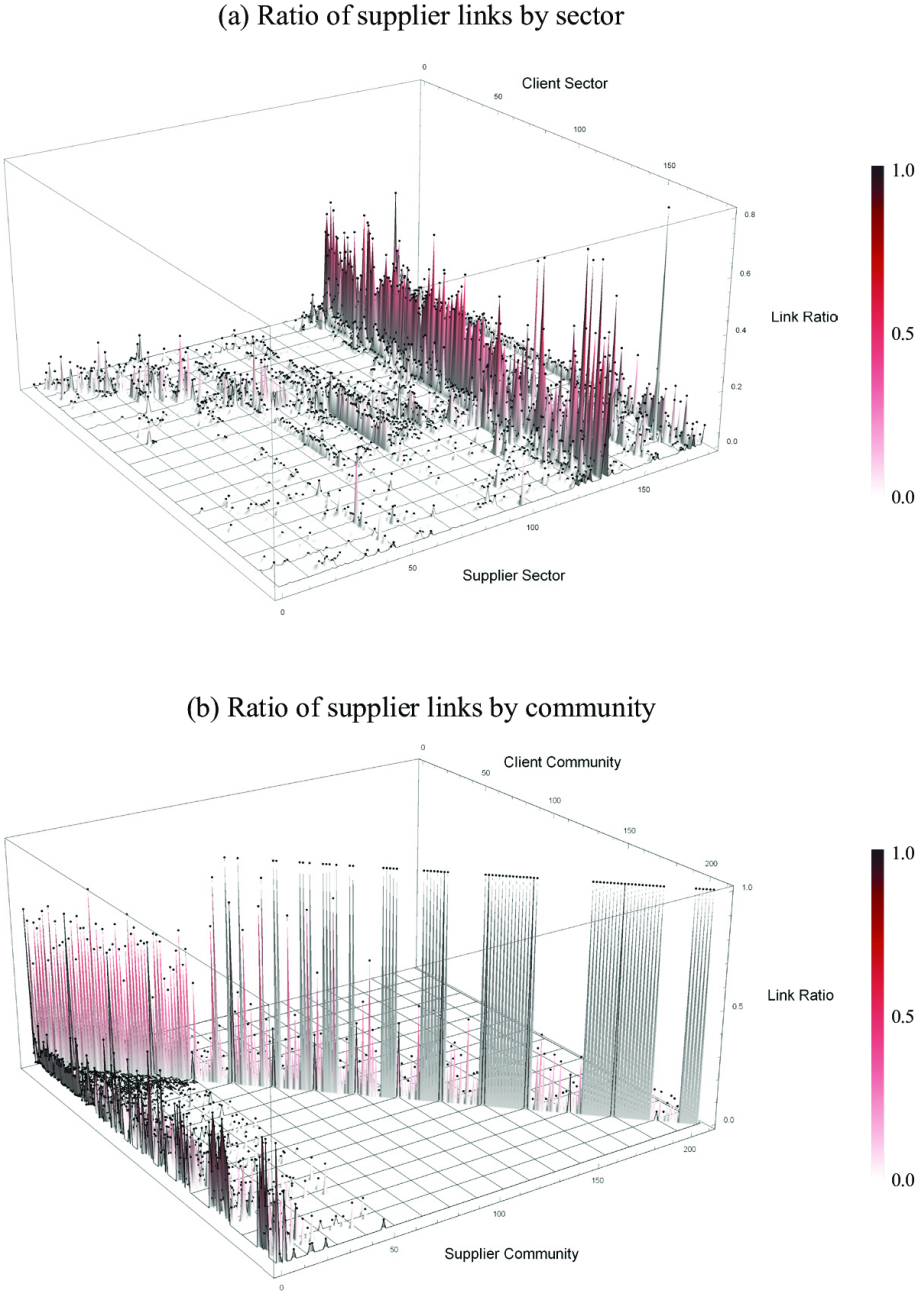
Density of links over intergroups. These figures show how many links the intergroups have. The top figure (a) shows the 3D plots of the industrial sectors. The bottom figure (b) shows the 3D plots of the communities.

First, we counted the number of links between the communities and the industrial sectors. Fig 14 shows the difference between these two groups. These figures correspond to matrices that show the number of links in row groups and column groups. Each element is divided by the sum of its row.

If the intra-links within the groups are dominant, then the diagonal elements of these matrices should have high density. As is shown in Fig 14, we can find the diagonal elements because the communities are denser than the other elements. However, the diagonal elements of the sectors do not have dense links. We see a vertical line in the matrix instead. The suppliers in the line include 5111: Wholesale and 5112: Retailing, and this result is natural because firms sell their products to industrial sectors. The overall ratio of intra-links, i.e., (the number of intra-group links)/(the number of all links) is 20.9% for industrial sectors and 63.3% for communities.

We can conclude that the detected communities in this paper explicitly illustrate the agglomeration of firms based on supply-chain networks rather than industrial sectors, which is more commonly used to categorize firms. This result also tells us that communities with densely connected firms consist of various industrial sectors, and they have their own economies, i.e., small universes.

In this paper, we do not weight the links of the network. However, obviously, each transaction has a value, and there is a diversity of transactions. We can estimate the weights by using the sales of the firms. If we have totally different results with the results we have obtained here, a further analysis might be necessary. However, the additional analyses based on weighting the links in the networks do not show any significant difference. The details of these results are shown in S1 Appendix: Intra-link density of the weighted links.

## Conclusion and discussion

We analyze the overall structure and hierarchical communities embedded in the production network of one million firms and five million links that represent trade relationships in Japan in 2016, with the aim of simulating the macro/micro level dynamics of the economy.

For the former, we find that the IN and OUT components (20% and 26% of the firms) form tight shells (semi-spheres) around the GSCC component, which we call a “walnut” structure rather than a “bow-tie” structure, which is well-known for representing web networks and other type of networks that have loose wings made of IN and OUT components.

For the latter, we use the Infomap method to detect a hierarchy that includes 5 layers of communities, of which most of the irreducible (those that do not have any lower level subcommunities) belong to the 2nd level. Furthermore, the size distribution of the 2nd level communities show clear power-law behavior at the large end. In addition to the large number of irreducible communities made primarily of GSCC components and those that exist in IN shells or Out shells, there is a fair number of communities made of IN and GSCC components, GSCC and OUT components, and even IN and OUT components. These communities are expected due to the walnut shape of the overall structure: IN and OUT components are not far from each other as they are in the bow-tie structure, but they form tight shells, whose ends are closely woven with each other. Furthermore, we examine the overexpression of the major communities in terms of industrial sectors and prefectures and find that they are not formed within a sector but span several sectors and prefectures. These communities have various shapes: in some cases, they are formed around goods and services related to a particular item, such as food. Sometimes these communities are made of small firms connected with a major hub such as a large construction company in a particular prefecture or a medical insurance agency.

These findings have major implications for the study of the macro economy: Consider an economic crisis. Once this crisis starts, whether it is due to a natural disaster in a particular region of a country or a major failure of a large company, it is expected that it initially affects the community in which this region or company is located. Then the effects of this crisis will spread to other neighboring communities. This analysis is very different from input-output analysis and is expected to be useful because an input-output analysis is based on the assumption that firms in the same sectors are well-connected with each other. In contrast, what we find is that the effects of a crisis will spread throughout communities rather than industries. The hierarchical community structure studied in this paper can be immediately applied to the analysis of large-scale modelling and simulation: the macro economy of a country or countries is an aggregation of products that economically affect the trade network as well as a multitude of networks of networks. Constructing models that span all the networks would be an interesting but exhaustive elaboration of this work. Instead, we may study one community at a time and then connect the results to obtain an overall picture. Research in this direction has already begun and will appear in the near future ([14, 31, 32]).

## Supporting information

S1 Appendix Appendix to the manuscript. (PDF)
