# Supplementary material for: Hierarchical communities in the walnut structure of the Japanese production network
Source: PLoS One. 2018 Aug 29;13(8):e0202739. doi: 10.1371/journal.pone.0202739 (PMC6114793; doi:10.1371/journal.pone.0202739)
Supplement: S1 Appendix — (PDF) [file pone.0202739.s001.pdf]

Appendix S1: Hierarchical communities in the walnut structure of the Japanese production network

DATA CLASSIFICATIONS

Table A lists the number of firms in 20 industrial sectors.

TABLE A. **Industrial sectors and firm distribution**

ID	Code	Sector	# Firms	%
1	A	Agriculture	9,841	0.92
2	B	Fisheries	1,211	0.11
3	C	Mining	1,268	0.12
4	D	Construction	357,199	33.51
5	E	Manufacturing	156,188	14.65
6	F	Electricity, Gas, Heat Supply & Water (*EGW*)	1,470	0.14
7	G	*Information* & Communications	26,539	2.49
8	H	*Transport* & Postal	36,736	3.45
9	I	Wholesale & *Retail* Trade	254,251	23.85
10	J	*Finance* & Insurance	7,506	0.70
11	K	Real Estate	41,837	3.92
12	L	*Scientific Research*, Professional & Technical Services	42,030	3.94
13	M	*Accommodations*, Eating/Drinking Services	17,322	1.62
14	N	*Living-related*/Personal & Amusement Services	17,365	1.63
15	O	*Education*, Learning Support	4,655	0.44
16	P	*Medical*, Health Care & Welfare	30,154	2.83
17	Q	Compound Services	6,472	0.61
18	R	Other Services	52,190	4.90
19	S	Government	1,803	0.17
20	T	Unable to classify	0	0.0

The number of firms classified by industrial sectors, which is based on the Japan Standard Industrial Classification. The words in italics are abbreviated in the main text.

Table B lists the number of firms in 8 regions and city of Tokyo, Japan, which are illustrated in Figure A.

TABLE B. **Regional areas and firm distribution**

id	region	#firms	%
1	Hokkaido	54,423	5.11
2	Tohoku	87,374	8.20
3	Kanto	187,186	17.56
4	Tokyo	146,614	13.75
5	Chubu	196,477	18.43
6	Kansai	168,701	15.83
7	Chugoku	69,312	6.50
8	Shikoku	40,397	3.79
9	Kyusyu-Okinawa	115,553	10.84

The number of firms in each regional area is determined by the geographical location of the main office of the firm. “Kanto” means “Kanto less Tokyo”, as greater “Tokyo” is in the “Kanto” region.

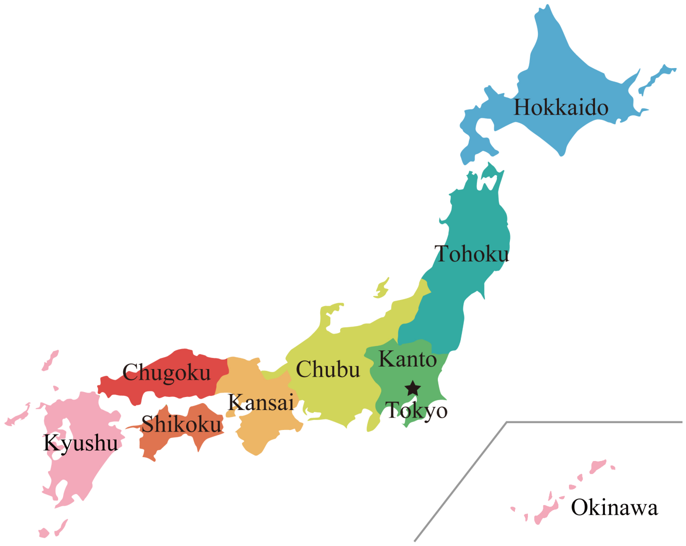

FIG. A. **Eight regions and Tokyo, Japan.**

COMPARISON OF THE COMMUNITY STRUCTURE BASED ON MODULARITY MAXIMIZATION

Fig B shows the complementary cumulative distribution of the community size at the top level, which is compared with the corresponding result obtained by the modularity maximization method. The two distributions are quite similar, indicating that the two community structures are similar. The distribution for modularity maximization is obtained for a undirected network, that is, by ignoring the direction of the links. We also conducted directed modularity analysis, but the result does not differ much from those reported above.

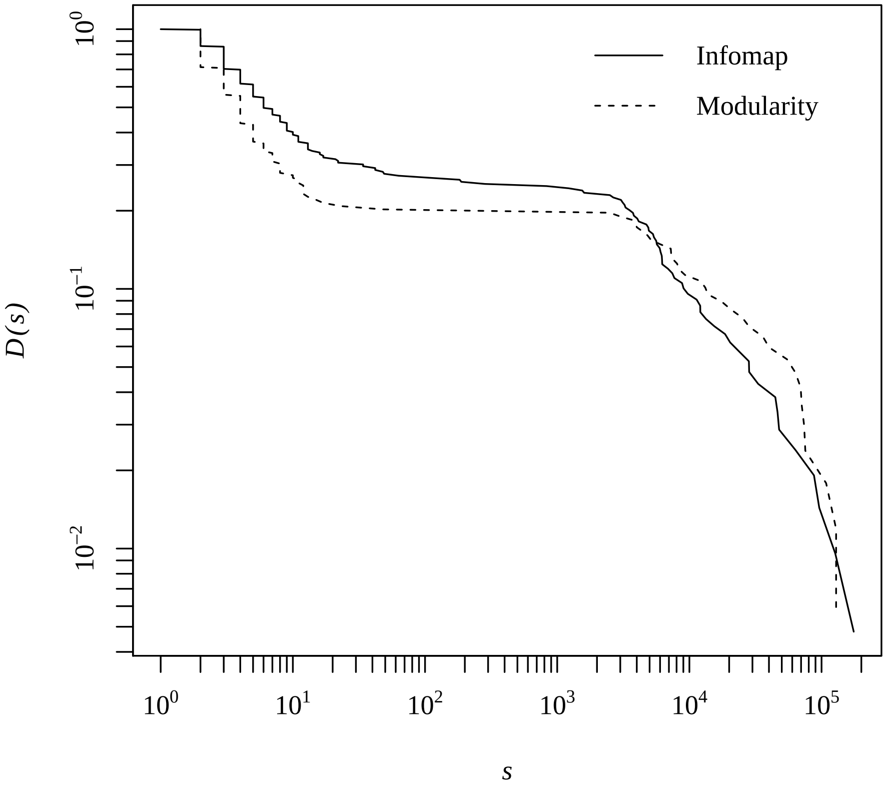

FIG. B. **The complementary cumulative distribution function $D(s)$ of communities with size s at the top modular level.**

One can quantify the similarity between the 1st level of the community structure obtained with the map equation and that obtained with modularity in terms of the Rand index [1], which is a measure of similarity between two data clusterings. The adjusted Rand index, in which the coincidental chance that a clustering of a pair of nodes is identical, is subtracted from the original index and calculated as 0.325. This large value indicates that the two partitions resemble each other significantly.

In Table C, a more detailed comparison between the two community structures is made, that is, a community by community comparison is made using the Jaccard index [2], which measures the similarity between two sets. We see that there is remarkable one-to-one correspondence between the two partitions. The major communities of the map equation, up to the 7th largest in Table C, have counterparts in the partition with modularity.

TABLE C. **Jaccard index between the major communities of the top level obtained with modularity and those obtained with the hierarchical map equation**

	1	2	3	4	5	6	7	8	9	10
1	0.02	0.64	0.01	0.01	0.01	0.01	0.01	0.02	0.01	0.01
2	0.41	0.01	0.01	0.03	0.01	0.00	0.00	0.05	0.01	0.01
3	0.04	0.01	0.31	0.01	0.01	0.00	0.00	0.03	0.00	0.00
4	0.02	0.03	0.01	0.01	0.11	0.09	0.01	0.12	0.01	0.20
5	0.03	0.00	0.14	0.01	0.00	0.00	0.00	0.02	0.00	0.00
6	0.03	0.03	0.01	0.28	0.01	0.00	0.01	0.03	0.04	0.02
7	0.05	0.01	0.02	0.02	0.32	0.01	0.01	0.01	0.01	0.03
8	0.12	0.01	0.05	0.01	0.02	0.00	0.00	0.02	0.02	0.00
9	0.01	0.03	0.01	0.01	0.01	0.01	0.50	0.02	0.00	0.01
10	0.01	0.01	0.00	0.00	0.01	0.39	0.06	0.01	0.01	0.00

The 10 largest communities of the map equation are aligned in the horizontal direction and those of modularity, in the vertical direction. Note that the Jaccard index takes 1/3 for two sets of equal size, and 50% of the elements overlap.

OVEREXPRESSIONS: LEVEL 1

In the main text, we present the overexpression of 20 sector divisions and 9 regions in the selected large communities. Here, we further expose the overexpression of 99 sectors and 47 prefectures in the following communities.

Rank: 1

Over-expression of prefectures: Ibaraki; Gunma; Saitama; Chiba; Tokyo; Kanagawa; Nagano; Shizuoka; Aichi; Mie; Osaka; Hyogo; and Hiroshima

Over-expression of sectors: Equipment installation work; Manufacture of plastic products, except otherwise classified; Manufacture of rubber products; Manufacture of iron and steel; Manufacture of non-ferrous metals and products; Manufacture of fabricated metal products; Manufacture of general purpose machinery; Manufacture of production machinery; Manufacture of business oriented machinery; Electronic parts, devices and electronic circuits; Manufacture of electrical machinery, equipment and supplies; Manufacture of information and communication electronics equipment; Manufacture of transportation equipment; Production, transmission and distribution of electricity; Heat supply; Wholesale trade (general merchandise); Wholesale trade (building materials, minerals and metals, etc); Wholesale trade (machinery and equipment); Retail trade (machinery and equipment); Machine, etc.; and repair services, except those that are otherwise classified

Rank: 2

Overexpression of prefectures: Hokkaido; Aomori; Iwate; Miyagi; Akita; Yamagata; Fukushima; Niigata; Yamanashi; Nagano; Shizuoka; Tottori; Shimane; Tokushima; Kagawa; Ehime; Kochi; Saga; Nagasaki; Kumamoto; Oita; Miyazaki; and Kagoshima

Overexpression of sectors: Agriculture; Fisheries, except Aquaculture; Aquaculture; Manufacture of food; Manufacture of beverages, tobacco and feed; Railway transport; Warehousing; Wholesale trade (general merchandise); Wholesale trade (food and beverages); Miscellaneous wholesale trade; Retail trade (general merchandise); Retail trade (food and beverage); Non-store retailers; Financial institutions for cooperative organizations; Non-deposit money corporations, including lending and credit card businesses; Real estate lessors and managers; Accommodations; Eating and drinking places; Food take out and delivery services; Miscellaneous living-related and personal services; Services for amusement and recreation; Social insurance, social welfare and care services; Cooperative associations and not elsewhere classified; and Miscellaneous services

Rank:3

Overexpression of prefectures: Ibaraki; Saitama; Chiba; Tokyo; Kanagawa; and Osaka

Overexpression of sectors: Mining and quarrying of stone; Construction work, general including public and private construction work; Construction work by specialist contractors, except equipment installation work; Equipment installation work; Manufacture of ceramic, stone and clay products; Collection, purification and distribution of water and sewage collection, processing and disposal; Railway transport; Financial auxiliaries; Real estate agencies; Real estate lessors and managers; Goods rental and leasing; Technical services and not elsewhere classified; Social insurance, social welfare and care services; Automobile maintenance services; Political, business and cultural organizations; and Local government services

Rank: 4

Overexpression of prefectures: Aomori; Iwate; Miyagi; Akita; Fukushima; Ibaraki; Tochigi; Gunma; Saitama; Chiba; Kanagawa; Ishikawa; Shizuoka; Aichi; Mie; and Saga

Overexpression of sectors: Manufacture of petroleum and coal products; Manufacture of transportation equipment; Road Passenger transport; Road freight transport; Water transport; Warehousing; Services incidental to transport; Wholesale trade (machinery and equipment); Retail trade (machinery and equipment); Miscellaneous retail trade; Insurance institutions, including insurance agents brokers and services; Goods rental and leasing; School education; Waste disposal business; and Automobile maintenance services

Rank: 5

Over-expression of prefectures: Tokyo; Kanagawa; and Osaka

Over-expression of sectors: Printing and allied industries; Manufacture of business oriented machinery; Electronic parts, devices and electronic circuits; Manufacture of information and communication electronics equipment; Miscellaneous manufacturing industries; Communications; Brodcasting; Information services; Services incidental to the internet; Video picture information, sound information, and character information production and distribution; Air transport; Wholesale trade (machinery and equipment); Retail trade (machinery and equipment); Miscellaneous retail trade; Non-store retailers; Banking; Financial institutions for cooperative organizations; Non-deposit money corporations, including lending and credit card businesses; Financial products transaction dealers and futures commodity dealers; Financial auxiliaries; Real estate lessors and managers; Goods rental and leasing; Professional services and

not elsewhere classified; Advertising; Technical services and not elsewhere classified; Miscellaneous living-related and personal services; Services for amusement and recreation; School education; Miscellaneous education and learning support; Employment and worker dispatching services; Miscellaneous business services; and Political, business and cultural organizations

OVEREXPRESSIONS: SECOND MODULAR LEVEL

A short summary of the overexpression of 20 sector divisions and 9 regions in the 2nd level communities is provided in Tables D and E.

TABLE D. **Overexpressions of the 2nd level communities**

Index	Size	Rank	Region	Sector	IN	GSCC	OUT
1:1	2,618	6	Hokkaido (0.04); Tohoku (0.10); Chugoku (0.09); Shikoku (0.05); Kyusyu-Okinawa (0.12)	Retail (0.85)	0.03	0.10	0.86
1:2	1,430	16	Kansai (0.32)	Transport (0.08); Retail (0.31)	0.28	0.68	0.03
1:3	1,132	25	Hokkaido (0.04); Tohoku (0.12); Chugoku (0.11); Shikoku (0.05); Kyusyu-Okinawa (0.11)	Retail (0.77)	0.06	0.14	0.79
1:4	863	38	Hokkaido (0.06); Tohoku (0.11); Shikoku (0.04)	Retail (0.82)	0.05	0.12	0.83
1:5	854	39	Hokkaido (0.04); Chugoku (0.10); Kyusyu-Okinawa (0.13)	Retail (0.43); Accommodations (0.03)	0.11	0.59	0.29
2:1	2,474	8	Tokyo (0.18); Chubu (0.23)	Information (0.02); Transport (0.13); Accommodations (0.19); Living-related (0.49); Education (0.009); Services N.E.C. (0.04)	0.17	0.44	0.38
2:2	1,200	23	Tokyo (0.45); Kansai (0.22)	Manufacturing (0.23); Retail (0.55); Scientific research (0.04)	0.53	0.37	0.09
2:3	1,121	27	Kanto (0.20); Chubu (0.24); Kansai (0.18)	Manufacturing (0.26); Retail (0.60)	0.09	0.56	0.35
2:4	1,022	28	Kanto (0.22); Tokyo (0.25), Kansai (0.26)	Retail (0.72)	0.12	0.34	0.53
2:5	1,010	29	Kanto (0.33)	Agriculture (0.13); Retail (0.67)	0.10	0.49	0.40
3:1	2,173	12	Hokkaido (0.02); Tohoku (0.05); Tokyo (0.33); Chubu (0.09); Chugoku (0.04); Shikoku (0.02); Kyusyu-Okinawa (0.05)	Manufacturing (0.05); Scientific research (0.09), Services N.E.C. (0.06)	0.60	0.36	0.05
3:2	834	41	Tokyo (0.55)	Informations (0.02); Real estate(0.73); Scientific research (0.09)	0.30	0.25	0.40
3:3	776	46	Tokyo (0.97)	Scientific research(0.12); Medical (0.32); Services, N.E.C. (0.22); Government (0.02)	0.70	0.12	0.11
3:4	740	53	Tohoku (0.08); Chubu (0.16); Chugoku (0.06); Shikoku (0.02); Kyusyu-Okinawa (0.09)	Construction (0.78)	0.68	0.26	0.06
3:5	547	87	Hokkaido (0.02); Tohoku(0.06); Chubu (0.20); Chugoku (0.08); Shikoku (0.04); Kyusyu-Okinawa (0.12)	Construction (0.84)	0.83	0.12	0.05

The overexpression in terms of regions and sector-divisions for the five largest communities at the 2nd level. "Rank" refers to the rank among all the 2nd level subcommunities. The percentage of nodes having a particular attribute is indicated in parentheses.

In addition, we present the overexpression of 99 sectors and 47 prefectures in the following subcommunities.

TABLE E. **Overexpressions of the 2nd level communities, continued**

Index	Size	Rank	Region	Sector	IN	GSCC	OUT
4:1	7,843	1	Hokkaido (0.06); Tokyo (0.20); Shikoku(0.04)	Information (0.02); Finance (0.54); Real estate (0.12); Scientific research (0.03)	0.09	0.13	0.77
4:2	2,747	5	Hokkaido (0.05); Kanto (0.27); Chubu (0.23)	Retail (0.87)	0.04	0.34	0.61
4:3	2,249	11	Shikoku (0.05); Kyusyu-Okinawa (0.13)	Retail (0.87)	0.03	0.12	0.85
4:4	1,416	17	Hokkaido (0.06); Shikoku (0.04)	Retail (0.88)	0.03	0.13	0.83
4:5	1,149	24	Kansai (0.96)	Retail(0.46); Services, N.E.C. (0.36)	0.06	0.43	0.51
5:1	2,996	4	Hokkaido (0.05); Tohoku (0.07); Kanto (0.17); Chubu (0.20); Kansai (0.16); Chugoku (0.06); Kyusyu-Okinawa (0.10)	Living-related (0.54)	0.09	0.23	0.62
5:2	1,248	22	Hokkaido (0.05); Tohoku (0.07); Kanto (0.22); Chubu (0.18)	Transport(0.04); Retail(0.62)	0.11	0.22	0.66
5:3	1,127	26	Chubu (0.28)	Manufacturing(0.12); Retail (0.62), Education (0.09)	0.09	0.29	0.61
5:4	832	42	Kansai (0.17)	Retail (0.33); Real estate (0.10); Living-related(0.27)	0.16	0.39	0.43
5:5	750	50	Hokkaido (0.05); Shikoku (0.04)	Information (0.32); Retail (0.39)	0.26	0.37	0.36

The overexpression in terms of regions and sector-divisions in the five largest communities at the 2nd level, continued. "Rank" refers to the rank among all the 2nd level subcommunities. The percentage of nodes having a particular attribute is indicated in parentheses.

Five largest subcommunities

Rank: 1

Overexpression of prefectures: Hokkaido; Tokyo; Fukui; Yamanashi; Tokushima; Kagawa; and Ehime

Overexpression of sectors: Information services; Financial products transaction dealers and futures commodity dealers; Insurance institutions, including insurance agents brokers and services; Real estate agencies; Real estate lessors and managers; Professional services, not elsewhere classified; and Miscellaneous living-related and personal services

Rank: 2

Overexpression of prefectures: Ibaraki; Tochigi; Gunma; Saitama; Chiba; Kanagawa; Nagano; Gifu; Shizuoka; Aichi; Mie; Osaka; Hyogo; Nara; and Wakayama

Overexpression of sectors: Miscellaneous retail trade and Medical and other health services

Rank: 3

Overexpression of prefectures: Iwate; Tochigi; Kanagawa; Kyoto; Hiroshima; Fukuoka; Miyazaki; and Kagoshima

Overexpression of sectors: Medical and other health services

Rank: 4

Overexpression of prefectures: Hokkaido; Iwate; Tochigi; Gunma; Gifu; Aichi; Kyoto; Fukuoka; and Kagoshima

Overexpression of sectors: Manufacture of plastic products, except otherwise classified; Manufacture of business oriented machinery; Wholesale trade (machinery and equipment); Miscellaneous wholesale trade; Real estate lessors and managers; and Services for amusement and recreation

Rank: 5

Overexpression of prefectures: Hokkaido; Chiba; Kanagawa; Gifu; and Aichi

Overexpression of sectors: Wholesale trade (general merchandise); Wholesale trade (machinery and equipment); and Retail trade (machinery and equipment)

Five largest subcommunities of the largest community:

Rank: 6

Overexpression of prefectures: Hokkaido; Aomori; Iwate; Akita; Niigata; Toyama; Ishikawa; Fukui; Okayama; Yamaguchi; Tokushima; Kagawa; Ehime; Kochi; Kumamoto; Oita; and Kagoshima

Overexpression of sectors: Retail trade (machinery and equipment)

Rank: 16

Overexpression of prefectures: Chiba; Osaka; and Wakayama

Over-expression of sectors: Construction work by specialist contractors, except equipment installation work; Manufacture of textile products; Manufacture of iron and steel; Road freight transport; Warehousing; Services incidental to transport; Wholesale trade (textile and apparel); Wholesale trade (building materials, minerals and metals, etc); and Waste disposal business

Rank: 25

Overexpression of prefectures: Hokkaido; Aomori; Akita; Toyama; Yamaguchi; Tokushima; Kochi; Saga; and Okinawa

Overexpression of sectors: Retail trade (machinery and equipment)

Rank: 38

Overexpression of prefectures: Hokkaido; Iwate; Akita; Niigata; Toyama; and Fukui

Overexpression of sectors: Retail trade (machinery and equipment)

Rank: 39

Overexpression of prefectures: Hokkaido; Shimane; Miyazaki; Kagoshima; and Okinawa

Overexpression of sectors: Manufacture of furniture and fixtures; Wholesale trade (machinery and equipment); Miscellaneous wholesale trade; and Eating and drinking places

Five largest subcommunities of the 2nd largest community:

Rank: 8

Overexpression of prefectures: Tokyo; Yamanashi; Nagano; and Okinawa

Overexpression of sectors: Road passenger transport; Water transport; Accommodations; Miscellaneous living-related and personal services; Miscellaneous education and learning support; Employment and worker dispatching services; and Political, business and cultural organizations

Rank: 23

Overexpression of prefectures: Tokyo; Kanagawa; Kyoto; and Osaka

Overexpression of sectors: Manufacture of textile products; Manufacture of furniture and fixtures; Manufacture of leather tanning, leather products and fur skins; Miscellaneous manufacturing industries; Wholesale trade (general merchandise); Wholesale trade (textile and apparel); Miscellaneous wholesale trade; Retail trade (general merchandise); and Retail trade (woven fabrics, apparel, apparel accessories and notions)

Rank: 27

Overexpression of prefectures: Saitama; Aichi; and Osaka

Overexpression of sectors: Manufacture of food; Manufacture of production machinery; and Wholesale trade (food and beverages)

Rank: 28

Overexpression of prefectures: Saitama; Tokyo; Kanagawa; Kyoto; Osaka; and Hyogo

Overexpression of sectors: Manufacture of beverages, tobacco and feed; Wholesale trade (food and beverages); Retail trade (general merchandise); and Retail trade (food and beverage)

Rank: 29

Overexpression of prefectures: Ibaraki; Tochigi; Gunma; and Chiba

Overexpression of sectors: Agriculture; Forestry; Construction work, general including public and private construction work; Manufacture of chemical and allied products; Miscellaneous wholesale trade; and Miscellaneous retail trade

Five largest subcommunities of the 3rd largest community:

Rank: 12

Overexpression of prefectures: Hokkaido; Miyagi; Tokyo; Aichi; Hiroshima; Kagawa; and Fukuoka

Overexpression of sectors: Construction work by specialist contractors, except equipment installation work; Manufacture of fabricated metal products; Goods rental and leasing; Technical services, not elsewhere classified; Employment and worker dispatching services; and Miscellaneous business services

Rank: 41

Overexpression of prefectures: Tokyo and Osaka

Overexpression of sectors: Real estate agencies; Real estate lessors and managers; Professional services, not elsewhere classified; and Advertising

Rank: 46

Overexpression of prefectures: Tokyo

Overexpression of sectors: Professional services, not elsewhere classified; Technical services, not elsewhere classified; Social insurance, social welfare and care services; Political, business and cultural organizations; and Local government services

Rank: 53

Overexpression of prefectures: Iwate; Akita; Tochigi; Niigata; Ishikawa; Nagano; Shizuoka; Aichi; Mie; Nara; Wakayama; Okayama; Hiroshima; Yamaguchi; Kagawa; Fukuoka; Nagasaki; and Oita

Overexpression of sectors: Construction work, general including public and private construction work; and Construction work by specialist contractors, except equipment installation work

Rank: 87

Overexpression of prefectures: Miyagi; Tochigi; Niigata; Toyama; Gifu; Shizuoka; Aichi; Mie; Tottori; Shimane; Hiroshima; Tokushima; Kagawa; Fukuoka; Saga; Oita; and Okinawa

Overexpression of sectors: Construction work, general including public and private construction work; and Construction work by specialist contractor, except equipment installation work

Five largest subcommunities of the 4th largest community:

Rank: 1

Overexpression of prefectures: Hokkaido; Tokyo; Fukui; Yamanashi; Tokushima; Kagawa; and Ehime

Overexpression of sectors: Information services; Financial products transaction dealers and futures commodity dealers; Insurance institutions, including insurance agents brokers and services; Real estate agencies; Real estate lessors and managers; Professional services, not elsewhere classified; and Miscellaneous living-related and personal services

Rank: 5

Overexpression of prefectures: Hokkaido; Chiba; Kanagawa; Gifu; and Aichi

Overexpression of sectors: Wholesale trade, general merchandise; Wholesale trade (machinery and equipment); and Retail trade (machinery and equipment)

Rank: 11

Overexpression of prefectures: Saitama; Kanagawa;and Ehime

Overexpression of sectors: Retail trade (machinery and equipment)

Rank: 17

Overexpression of prefectures: Hokkaido and Osaka

Overexpression of sectors: Manufacture of transportation equipment; Wholesale trade (machinery and equipment); Retail trade (machinery and equipment); and Machine, etc. repair services, except otherwise classified

Rank: 24

Overexpression of prefectures: Osaka

Overexpression of sectors: Retail trade (woven fabrics, apparel, apparel accessories and notions) and Automobile maintenance services

Five largest subcommunities of the 5th largest community:

Rank: 4

Overexpression of prefectures: Hokkaido; Iwate; Tochigi; Gunma; Gifu; Aichi; Kyoto; Fukuoka; and Kagoshima

Overexpression of sectors: Manufacture of plastic products, except otherwise classified; Manufacture of business oriented machinery; Wholesale trade (machinery and equipment); Miscellaneous wholesale trade; Real estate lessors and managers; and Services for amusement and recreation

Rank: 22

Overexpression of prefectures: Hokkaido; Tochigi; Gunma; Chiba; Yamanashi; and Tokushima

Overexpression of sectors: Road freight transport; Miscellaneous retail trade; and Advertising

Rank: 26

Overexpression of prefectures: Nagano; Shizuoka; Aichi; and Mie

Overexpression of sectors: Manufacture of lumber and wood products, except furniture; Miscellaneous manufacturing industries; Miscellaneous wholesale trade; Miscellaneous retail trade; Miscellaneous education and learning support; and Machine, etc. repair services, except otherwise classified

Rank: 42

Overexpression of prefectures: Gifu and Osaka

Overexpression of sectors: Manufacture of business oriented machinery; Miscellaneous manufacturing industries; Miscellaneous wholesale trade; Miscellaneous retail trade; Goods rental and leasing; and Services for amusement and recreation

Rank: 50

Overexpression of prefectures: Kagoshima

Overexpression of sectors: Communications; Services incidental to the Internet;and Retail trade (machinery and equipment)

INTRA-LINK DENSITY OF WEIGHTED LINKS

Fig 13 of the main text in the Comparison of industrial sectors Section shows the matrixes that represent the number of inter oand intra links between groups. If we weight, i.e., using sales volume, the links and create the same matrixes for the weighted links, then the matrixes can further indicate the agglomerative behaviour of groups.

Although the TSR data contains data on supplier and client relationships, the sales volume for each relationship is not provided. Therefore, we artificially add sales volume using the method proposed in [3]. Each supplier's sales are proportionally divided into its clients' sales. Here, we ignore supplier's sales to final consumers, and the client's purchase from the supplier can be relatively estimated by using the sales of the clients as proxies.

Fig C provides the results. The visualization of the industrial sectors has denser connections for wholesale and retailing than that for the number of links shown in Fig 13 of the main text. In addition, the visualization of the communities has denser connections to communities in the left rows than that shown in Fig 13 of the main text. The overall ratio of internal sales volume, i.e., (the total volume of the intra-group links)/(the total volume of all links) is 14.3% for the industrial sectors and 55.9% for the communities.

As a result, we obtain weaker connections between the intra-groups for both industrial sectors and communities. The weaker intra-connections of these communities is understandable because we do not use weighted links to detect the communities. However, we do not expound on weighted community detection to simplify the discussion in this paper.

[1] Rand WM. Objective Criteria for the Evaluation of Clustering Methods. Journal of the American Statistical Association. 1971;66(336):846-850. doi:10.1080/01621459.1971.10482356.

[2] Jaccard P. tude comparative de la distribution florale dans une portion des Alpes et des Jura. Bulletin de la Socit Vaudoise des Sciences Naturelles. 1901;37:547-579.

[3] Inoue H, Todo Y. Propagation of negative shocks through firm networks: evidence from simulation on comprehensive supply-chain data. RIETI Discussion Papers. 2017;17-E-044:1-21.

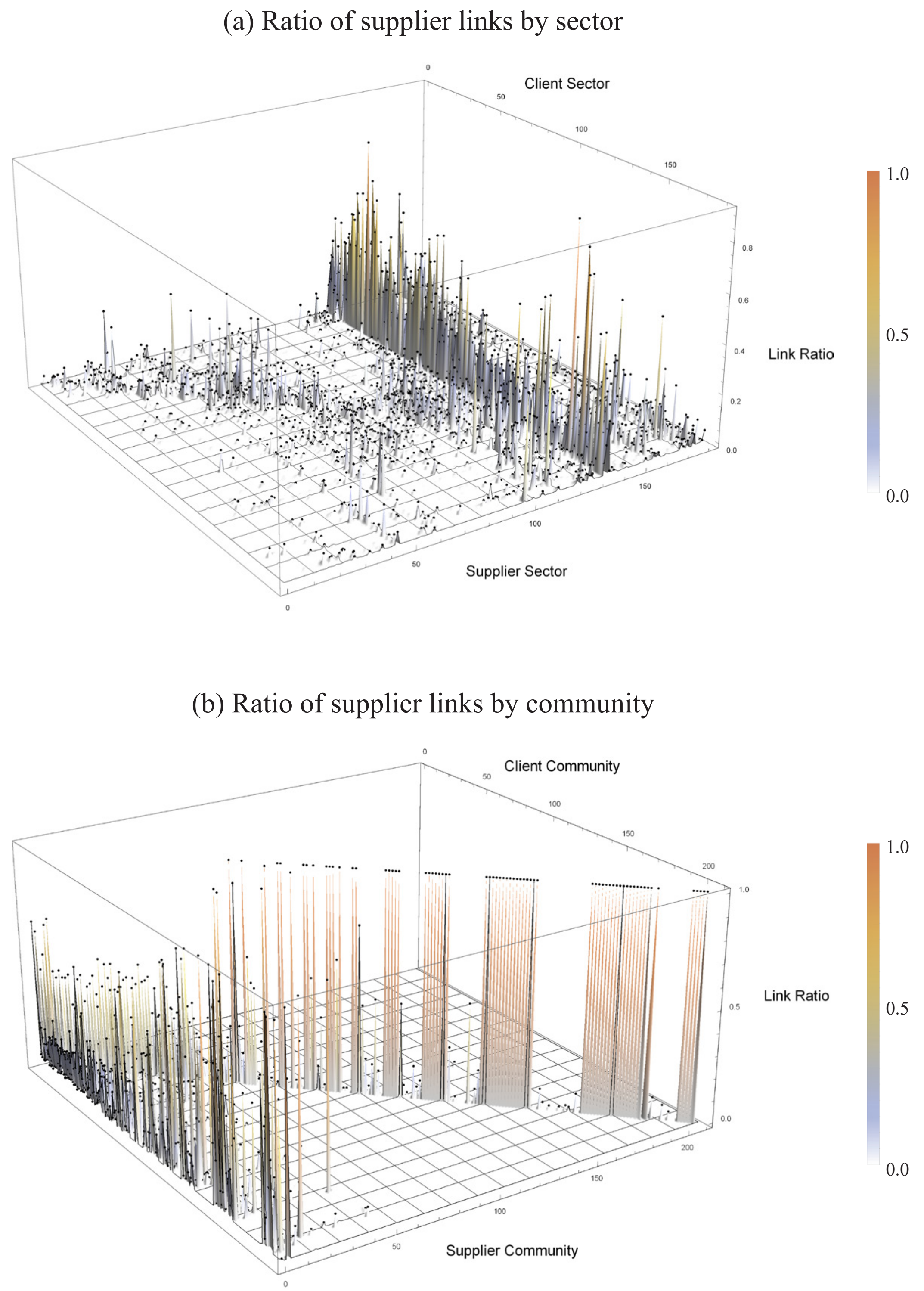

FIG. C. **Density of weighted links for the inter and intra-sectors of communities.** These figures show the sales volume of the intergroups. The top figure (a) shows the 3D plots of the industrial sectors. The bottom figure (b) shows the 3D plots of the communities.
